# Blockade of CB1 cannabinoid receptor alters gut microbiota and attenuates inflammation and diet-induced obesity

**DOI:** 10.1038/s41598-017-15154-6

**Published:** 2017-11-15

**Authors:** Pegah Mehrpouya-Bahrami, Kumaraswamy Naidu Chitrala, Mitra S. Ganewatta, Chuanbing Tang, E. Angela Murphy, Reilly T. Enos, Kandy T. Velazquez, Jamie McCellan, Mitzi Nagarkatti, Prakash Nagarkatti

**Affiliations:** 1 Department of Pathology, Microbiology, and Immunology, School of Medicine, Columbia, SC USA; 2 Department of Chemistry and Biochemistry, University of South Carolina, Columbia, SC USA

## Abstract

Obesity is characterized by chronic low-grade, systemic inflammation, altered gut microbiota, and gut barrier disruption. Additionally, obesity is associated with increased activity of endocannabinoid system (eCB). However, the clear connection between gut microbiota and the eCB system in the regulation of energy homeostasis and adipose tissue inflammation and metabolism, remains to be established. We investigated the effect of treatment of mice with a cannabinoid receptor 1 (CB1) antagonist on Diet-Induced Obesity (DIO), specifically whether such a treatment that blocks endocannabinoid activity can induce changes in gut microbiota and anti-inflammatory state in adipose tissue. Blockade of CB1 attenuated DIO, inflammatory cytokines and trafficking of M1 macrophages into adipose tissue. Decreased inflammatory tone was associated with a lower intestinal permeability and decreased metabolic endotoxemia as evidenced by reduced plasma LPS level, and improved hyperglycemia and insulin resistance. 16S rRNA metagenomics sequencing revealed that CB1 blockade dramatically increased relative abundance of *Akkermansia muciniphila* and decreased *Lanchnospiraceae* and *Erysipelotrichaceae* in the gut. Together, the current study suggests that blocking of CB1 ameliorates Diet-Induced Obesity and metabolic disorder by modulating macrophage inflammatory mediators, and that this effect is associated with alterations in gut microbiota and their metabolites.

## Introduction

The gut microbiome is the key feature in maintaining the whole body energy balance by affecting the glucose metabolism and low-grade chronic inflammation associated with obesity. Previous studies have shown that obese mice had significant changes at phylum-level in their microbial community and fecal transfer from obese mice into gnotobiotic lean mice conferred many inflammatory features of diet-induced obesity to the recipients^1,2^. Correlation between progression of metabolic syndrome and alteration in the gut microbial community has been reported in mice with deletion of Toll-Like Receptor 5(TLR5) gene^3^. Fecal transfer from the Toll-Like Receptor 5 (TLR5) deficient mice to wild-type germ-free mice mimics multiple symptoms of metabolic disease in the recipient mice.

The microbial community of the gut consists of symbionts (beneficial), neutral (commensals) as well as detrimental (pathobionts) microorganisms. The homeostasis of these essential allies can modulate the host health and function^4^. The mutual interaction of gut microbiota and host immune system is necessary for maintaining their symbiotic relationship^5–7^. Gut immune system determines colonization of microbial community in gut and contributes to the interaction of host-microbiome^8^.

Metabolic endotoxemia and inflammation in obese mice may result from the chronically higher levels of lipopolysaccharide (LPS) and pro-inflammatory cytokines^9^. Higher intake of saturated fat leads to the disruption of multi-layered mucus structures and tight junctions in the gut, resulting in the permeability of gut barrier and consequent leakage of lipopolysaccharide (LPS) into circulation^10^. Lipopolysaccharide (LPS) absorption by gut enterocyte chylomicrons results in the robust release of systemic lipopolysaccharide (LPS), which is believed to contribute to inflammatory and metabolic disorder^11^. Macrophages are the first-line of target of lipopolysaccharide (LPS) and their retention in adipose tissue is implicated in the pathophysiology of diet-induced obesity and metabolic syndrome^12^.

Numerous studies have demonstrated that diet-induced obesity and associated-inflammatory disorders may result from dysregulation of endocannabinoid (eCB) system. Augmentation in endocannabinoid (eCB) levels in plasma and adipose tissue, as well as modulation of cannabinoid CB1 receptors, has been reported in obese individuals^13,14^. Alterations in gut endocannabinoid (eCB) system are implicated in the dysregulation of lipopolysaccharide (LPS) level, gut integrity disruption, chronic inflammatory state of the gut, and dysbiosis of gut microflora^15^. A previous study has shown that lipopolysaccharide (LPS) leads to dysregulation of endocannabinoid (eCB) system in macrophages^16^. Lipopolysaccharide (LPS) causes robust production of endogenous ligands of cannabinoid receptors, specifically Anandamide (arachidonylethanolamide, AEA) in adipose tissue macrophages, which contributes to exacerbation of chronic inflammation in visceral fat, hyperglycemia and insulin resistance^16^.

Numerous pharmacological, preclinical and clinical studies indicate that blockade of cannabinoid CB1 receptors can significantly improve obesity complications and multiple risk factors of metabolic syndrome^17–19^. However, the direct implications and the precise physiological role of cannabinoid CB1 receptor antagonist in the modulation of gut microbial communities in Diet-Induced Obesity (DIO) has not yet been fully determined. Herein, for the first time, we uncovered the changes in the gut microbial community in a mouse model of Diet-Induced Obesity (DIO) treated with the cannabinoid CB1 antagonist, SR41716A. To demonstrate the effect of SR141716A on gut microbiota beyond its effect on diet intake and weight loss, we included pair-feeding controls as well as body weight-matched controls. The current study provides compelling evidence that targeting the endocannabinoid (eCB) system in Diet-Induced Obesity (DIO) model by utilizing SR141716A as the cannabinoid CB1 receptor blocker, remodels the gut microbial colonization and subsequently leads to amelioration of pro-inflammatory macrophages and metabolic parameters.

## Results

### Effect of SR141716A on diet intake, body weight and body composition

**Figure 1:**
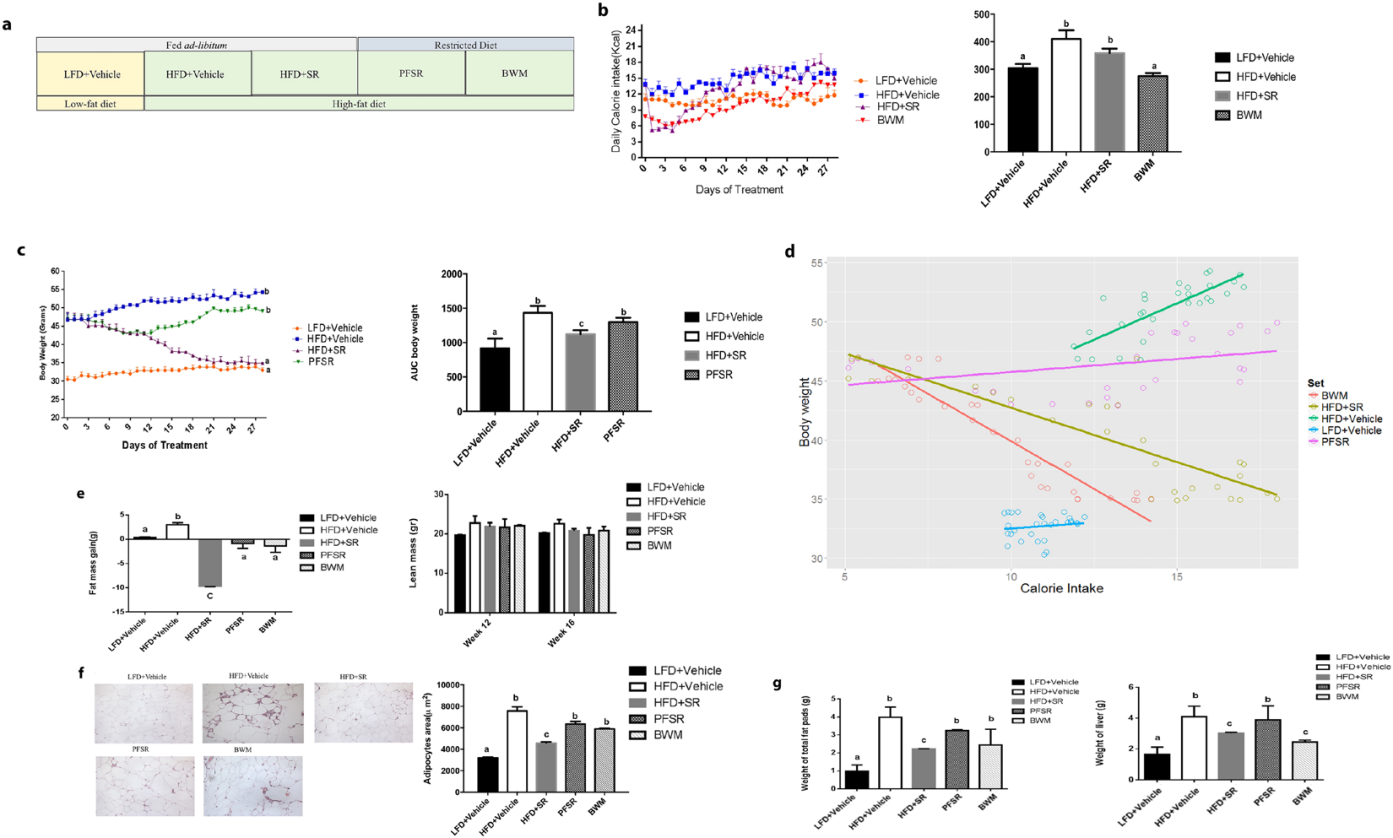
SR141716A causes transient reduction in diet intake and persistent weight loss when compared to vehicle-treated HFD fed control. (**a**) Diet-Induced Obesity (DIO) model was generated by feeding C57BL/6 J male mice with high-fat diet (HFD + Vehicle) whereas their lean, age-matched controls were fed low-fat diet (LFD + Vehicle). High Fat Diet-fed mice were treated with either SR141716A (10 mg/kg/day) (HFD + SR) or vehicle (0.1% Tween 80) (HFD + Vehicle) by daily oral gavage for 4 weeks starting at week 12. In order to assess the anti-inflammatory effect of SR141716A beyond its effect on calorie intake inhibition and weight loss in Diet-Induced Obesity (DIO) phenotype, pair-feeding was conducted in diet-intake matched controls (PFSR) and diet intake was adjusted in bodyweight-matched controls (BWM; n = 8–10 mice/group). (**b**) Daily energy intake during 4 week treatment with SR141716A in Diet-Induced Obesity (DIO) mice was recorded, Area Under the Curve (AUC) was calculated from the 5 replicated experiments. (**c**) Daily body weight of each group of mice is shown during the whole period of treatment; AUC was calculated from the 5 replicated experiments which were identical to the replicates in Fig. 1b. Area Under Curve (AUC) was calculated with Trapezoidal rule in R software. Generalized Estimating Equation (GEE) was performed to fit a repeated measurement logistic regression in SPSS. Data are shown as mean ± SD. Data with different superscript letters are significantly different (*P* < 0.05). (**d**) Pearson correlation between changes in body weight and caloric intake within different groups was assessed using R software. (**e**) Total fat mass gain and changes in lean mass was assessed at the baseline and after 4 weeks of treatment with Dual Energy X-ray absorptiometry (DEXA). Data are shown as mean ± SD. Data with different superscript letters are significantly different (*P* < 0.05). (**f**) The surface area of 100 adipocytes was determined and then averaged to represent mean adipocyte size for each mouse using ImageJ software (National Institutes of Health). Data are shown as mean $\pm$ SD. Data with different superscript letters are significantly different (*P* < 0.05) according to post hoc ANOVA one-way statistical analysis. (*n* = 10). (**g**) Weights of fat pads and livers were assessed at the end of the treatment.

Consistent with previous studies, treatment with SR141716A of mice with Diet-induced Obesity (DIO) (HFD + SR) transiently reduced calorie intake and induced weight loss when compared with vehicle-treated mice with Diet-Induced Obesity (DIO) (HFD + Vehicle) (Fig. 1b,c)^20^. To assess the effect of SR141716A beyond its effect on weight loss and calorie intake, pair-feeding was conducted in diet intake-matched control (PFSR), and food intake was adjusted in body weight-matched (BWM) controls (Fig. 1a). The temporary reduction of calorie intake in HFD + SR mice during the first week was diminished by day 9 of treatment, reaching similar intake as vehicle-treated Diet-Induced Obesity (DIO) group (HFD + Vehicle) (Fig. 1b). However, we noted consecutive weight loss in SR141716A-treated Diet-Induced Obesity (DIO) group (HFD + SR) through the end of treatment (Fig. 1c). To maintain the same body weight in body weight-matched (BWM) group as SR141716A-treated Diet-Induced Obesity (DIO) group (HFD + SR), their food was restricted to even lower intake level than SR141716A-treated Diet-Induced Obesity (DIO) group (HFD + SR) (Fig. 1b).

To examine the effect of SR141716A beyond its effect on calorie intake, the pair-fed control (PFSR) mice were fed with the same amount of high fat diet as consumed by the SR141716A-treated Diet-Induced Obesity (DIO) mice (HFD + SR). The weight loss pattern in pair-fed control (PFSR) group was similar to HFD + SR during the first two weeks of treatment, but then pair-fed control (PFSR) group started to gain weight, reaching body weight close to vehicle-treated Diet-Induced Obesity (DIO) mice (HFD + Vehicle), by the end of the treatment (Fig. 1c). Correlation between changes in body weight and caloric intake within different groups demonstrated consistent weight loss in SR141716A-treated Diet-Induced Obesity (DIO) group (HFD + SR) regardless of its high level of calorie intake, close to vehicle-treated Diet-Induced Obesity (DIO) group (HFD + Vehicle) (Fig. 1d).

Assessing body composition after four weeks of SR141716A intervention in Diet-Induced Obesity (DIO) mice (HFD + SR) showed a significant reduction in fat gain when compared to vehicle-treated Diet-Induced Obesity (DIO) mice (HFD + Vehicle), while there was no difference in lean mass (Fig. 1e). Because SR141716A-treated Diet-Induced Obesity (DIO) mice (HFD + SR) demonstrated less fat mass when compared to body weight-matched control (BWM), the data suggested that other factors are associated with the use of SR141716A besides its effect on calorie intake and weight loss (Fig. 1e). Lower fat mass within the SR141716A-treated group (HFD + SR) has been characterized with less adiposity. Assessing the area of the adipocytes demonstrated significant shrinkage in adipocytes of the SR141716A-treated Diet-Induced obesity (DIO) mice (HFD + SR) when compared to vehicle-treated Diet-Induced Obesity (DIO) mice (HFD + Vehicle), pair-fed controls (PFSR), and body weight-matched controls (BWM) (Fig. 1f). Furthermore, vehicle-treated Diet-Induced obesity mice (HFD + Vehicle) group demonstrated the presence of crown-like structures of macrophages surrounding the adipocytes, which were absent in other groups.

Adipose tissue fibrosis, in obese phenotype is associated with an increase in local inflammation. The Picrosirius red fibrillar collagens were interspersed among the adipocytes in vehicle-treated Diet-Induced obesity (HFD + Vehicle) group. SR141716A treatment in Diet-Induced Obesity (DIO) mice (HFD + SR) resulted in a significant suppression of adipose tissue fibrosis and consequently further reduction in local adipose tissue inflammation and dysfunction (Supplementary Figure 1a,b).

Lighter fat pad (mainly in the epididymal fat pad) in SR141716A-treated Diet-Induced Obesity (DIO) mice (HFD + SR) was associated with smaller liver weight when compared with vehicle-treated Diet-Induced obesity (DIO) (HFD + Vehicle), pair-fed Diet –induced Obesity (DIO) control (PFSR), and body weight-matched control (BWM) mice (Fig. 1g).

### Effect of SR141716A on systemic and local inflammation

**Figure 2:**
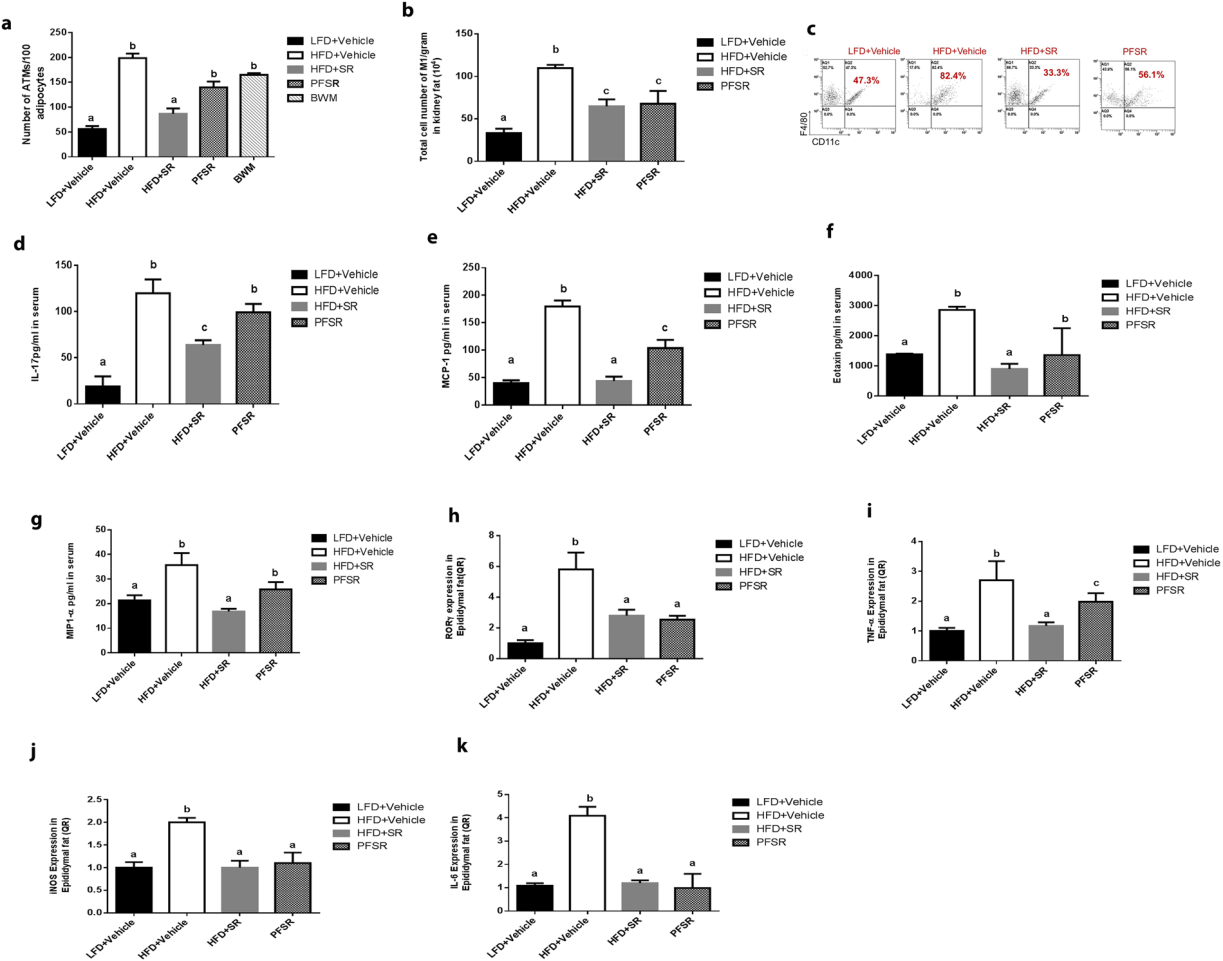
SR141716A attenuates local and systemic inflammation in diet-induced obesity. Experiments Diet-Induced Obesity (DIO) were set up as described in Fig. 1 legend. (**a**) Adipose Tissue Macrophages (ATMs) were quantified per 100 adipocytes by Spot Studio v1.0 Analysis Software. (**b**,**c**) Kidney fat was isolated from 10 mice in each group. The ratio (**b**) and total cell number (**c**) of kidney fat F4/80 and CD11c+ cells was studied. (**d**–**g**) Effect of SR141716A on plasma cytokine levels, (**d**) IL-17 levels (**e**) Monocyte chemoattractant protein-1(MCP-1) levels, (**f**) Eotaxin levels, and (**g**) Macrophage inflammatory protien-1**α** (MIP-1**α**) levels in plasma were quantified with multiplex immunoassays. (**h**,**k**) Effect of SR141716A treatment on the mRNA level of (**h**) RORγ, (**i**) TNF-𝛼, (**j**) iNOS, and (**k**) IL-6, in the epididymal adipose tissue was examined. Data shown as mean $\pm$ SD. Data with different superscript letters are significantly different (*P* < 0.05) according to post hoc ANOVA one-way statistical analysis. (*n* = 5 except LFD + Vehicle; *n* = 4).

Based on the active role of macrophages in the initiation of inflammation in adipose tissue, we examined the changes in macrophage population in adipose tissue. Intervention treatment of Diet-Induced Obesity (DIO) mice with SR141716A (HFD + SR) demonstrated significant reduction in the ratio of macrophages/adipocytes as compared with vehicle-treated Diet-Induced Obesity (DIO) (HFD + Vehicle) mice, pair-fed control(PFSR), and body weight matched-control (BWM) (Fig. 2a). Flow cytometric analysis of the subset of macrophages showed a significant reduction in both frequency and the absolute number of pro-inflammatory M1 macrophages with SR141716A treatment (HFD + SR) when compared to vehicle-treated Diet-Induced Obesity (DIO) controls (HFD + Vehicle) (Fig. 2b,c). The inflammatory profile was also assessed by examining chemokines and cytokines in the serum for systemic inflammation. Treatment of Diet-Induced obesity (DIO) mice with SR141716A (HFD + SR) led to lower level of IL-17, monocyte chemoattractant protein-1 (MCP-1), eotaxin, and macrophage inflammatory protein-1α (MIP-1α) when compared to vehicle-treated Diet-Induced Obesity (DIO) mice (HFD + Vehicle) (Fig. 2d–g). The same trend was seen with TNF-α, IL-6, RANTES, MIP-1β and MIP-2 but the differences were not significant. Changes in lipopolysaccharide (LPS) as a primary stimulator of macrophages has been demonstrated^21^.

We also investigated the inflammation profile of adipose tissue and colon locally, and to that end, the mRNA level of RORγ, TNF-α, iNOS, and IL-6 was quantified in adipose tissue (Fig. 2h,k). Overall, intervention treatment of Diet-Induced Obesity (DIO) mice with SR141716A (HFD + SR) led to the improvement of the inflammatory state of adipose tissue beyond its effect on diet restriction.

We also observed a significant increase in both the percentage and numbers of CD4 + GATA3 + Th2 cells (anti-inflammatory T cell subset) following treatment with SR141716A of Diet-Induced Obesity (DIO) mice (HFD + SR) in adipose tissue (Supplementary Figure 2a,b).

Myeloid Derived-Suppressor Cells (MDSC) that are GR-1 + CD11b + have been identified as potent anti-inflammatory cells. In the current study, we noted that Myeloid Derived-Suppressor Cells (MDSCs) were increased with SR141716A treatment in Diet-Induced Obesity (DIO) mice (HFD + SR) when compared to vehicle-treated Diet-Induced Obesity (DIO) (HFD + Vehicle) in adipose tissue (Supplementary Figure 2c,d). We also assessed the changes in blood Myeloid Derived-Suppressor Cells (MDSCs) and found a significant decrease in Myeloid Derived-Suppressor Cells (MDSCs) following SR141716A treatment (Supplementary Figure 2e), thereby suggesting that there may be increased migration of such cells into the adipose tissue following SR141716A treatment.

Differential analysis of complete blood count (CBC) revealed significant leukocytosis in Diet-Induced Obesity (DIO) mice (HFD + Vehicle) when compared to SR141716A-treated Diet-Induced Obesity (DIO) (HFD + SR) and lean (LFD + Vehicle) mice. Leukocytosis in Diet-Induced Obesity (DIO) mice was more pronounced in the neutrophil subpopulation, which is the first responder to an inflammatory signal. Our data suggested that treating Diet-Induced Obesity (DIO) mice with SR141716A reduces neutrophilic leukocytosis (Supplementary Table 1). Furthermore, SR141716A treatment balanced the increased level of hemoglobin and hematocrit (HCT%) in Diet-Induced Obesity (DIO) mice. (Supplementary Table 1). Collectively, our data suggested that intervention treatment of Diet-Induced Obesity (DIO) mice with SR141716A (HFD + SR) attenuates systemic and local inflammation.

### Effect of SR141716A on metabolic parameters

**Figure 3:**
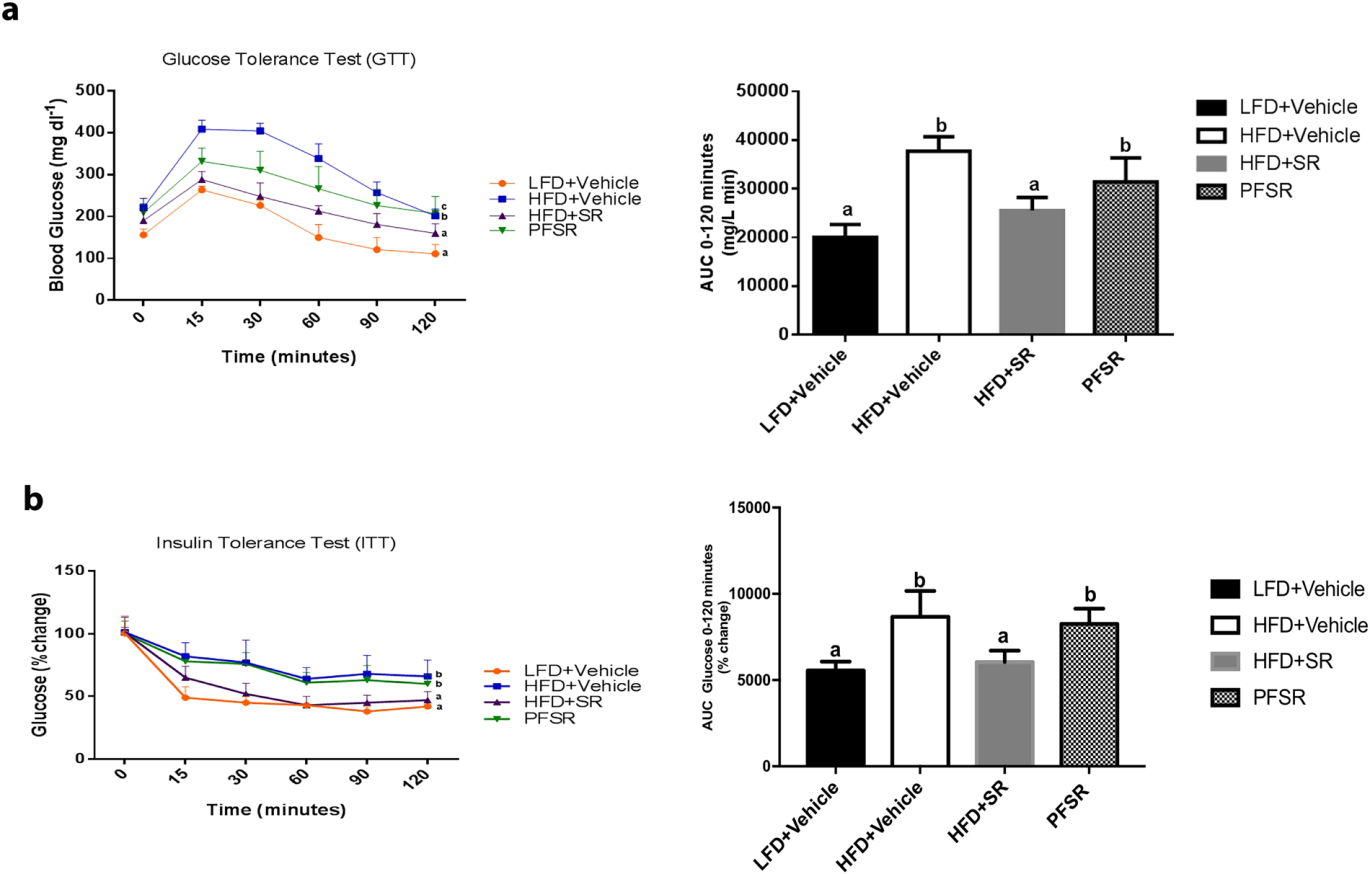
SR141716A ameliorates metabolic dysfunction in diet-induced obesity. Experiments Diet-Induced Obesity (DIO)were set up as described in Fig. 1 legend. (**a**) Glucose tolerance test (GTT) and (**b**) Insulin tolerance test (ITT) of mice fed LFD + Vehicle (n = 10), HFD + Vehicle (n = 10), HFD + SR (n = 9) and Pair-fed to SR141716A (PFSR) (n = 10). Each animal received by oral gavage 1.5 g/kg body mass of glucose (25% D-glucose). Blood glucose levels were determined after 15, 30, 60 and 120 minutes. Insulin-tolerance tests were carried out on un-fasted animals by i.p injection of 1.5 U/kg body mass of insulin. Blood glucose levels were detected after 15, 30, 60 and 120 minutes. Generalized Linear Mixed Model (GLMM) was performed to calculate p values for the repeated measures in SPSS. Mean Area Under the Curve (AUC) from triplicate experiments measured between 0–120 minutes after glucose (GTT) and insulin (ITT) load. AUC was assessed with Trapezoidal rule in R software. Data with different superscript letters are significantly different. GTT (*P* < 0.01), ITT (*P* < 0.05).

Glucose Tolerance Test (GTT) and Insulin Tolerance Test (ITT) showed remarkable improvement in metabolic parameters in Diet-Induced Obesity (DIO) mice when treated with SR141716A (HFD + SR) as compared with vehicle-treated Diet-Induced Obesity (DIO) (HFD + Vehicle) and pair-fed (PFSR) control mice (Fig. 3a,b). The intervention treatment of SR141716A in Diet-Induced Obesity (DIO) mice (HFD + SR) improved the serum metabolic parameters such as Fasting Blood Glucose (FBG), triglycerides (TGs), high-density lipoprotein (HDL), low-density lipoprotein (LDL), homeostatic model assessment (HOMA) index) when compared to vehicle-treated Diet-Induced Obesity (DIO) (HFD + Vehicle) and pair-fed to SR141716A (PFSR). (Supplementary Table 2). Additionally, SR141716A treatment reversed the increase in Free Fatty Acids in diet-induced obesity (Supplementary Figure 3).

### Effect of SR141716A on gut barrier integrity

**Figure 4:**
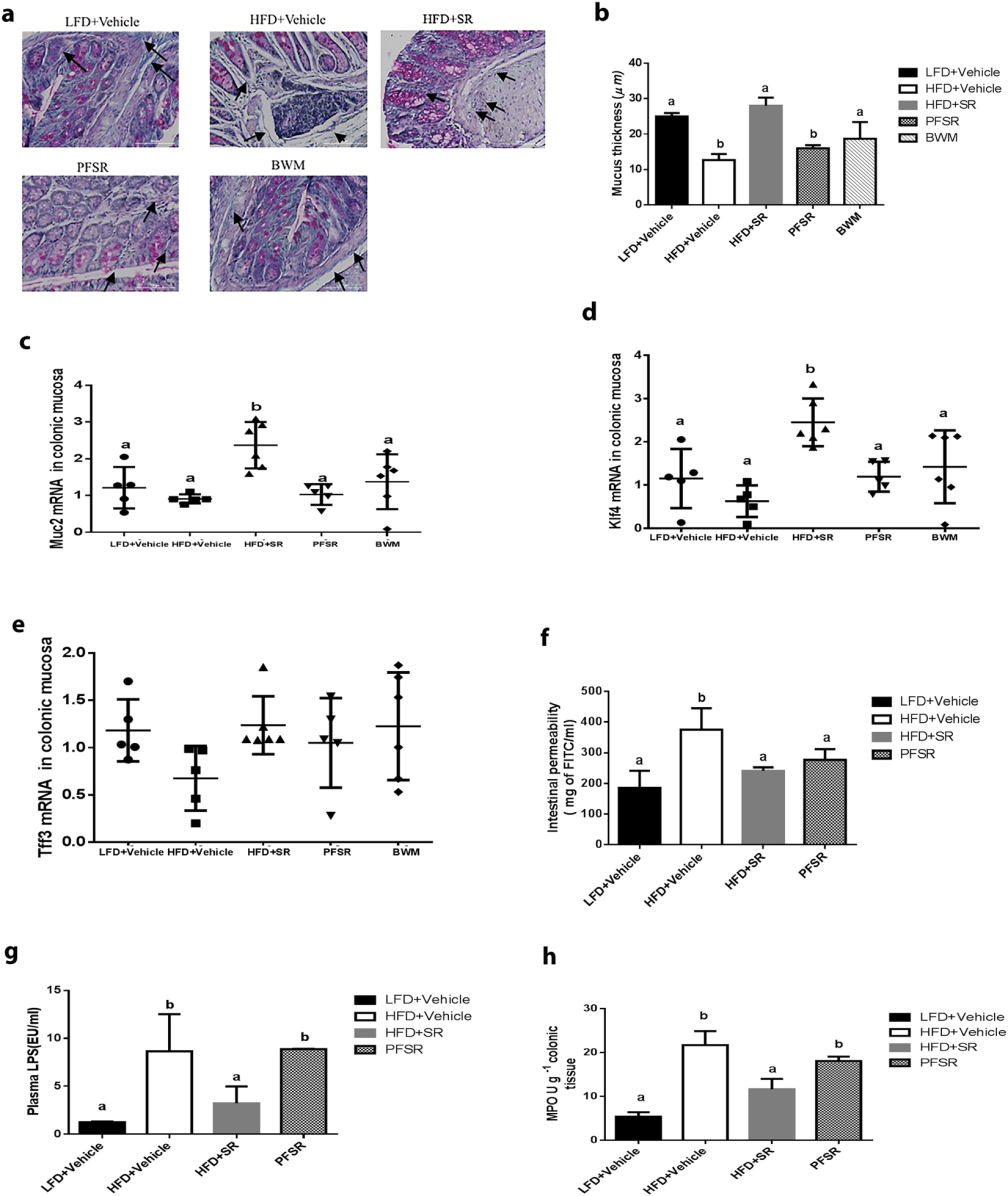
SR141716A restores gut barrier function in diet-induced obesity. Experiments Diet-Induced Obesity (DIO) were set up as described in Fig. 1 legend. (**a**) Representative Periodic Acid Schiff images were used for *in situ* mucus layer staining, scale bar,100 μm. (**b**) Thickness of the mucus layer measured by histological image analysis software MetaMorph (LFD + Vehicle, *n = 5;* HFD + Vehicle, *n = 5;* SR, *n = 6;* PFSR, *n = 5*; and BWM, *n = 6*). (**c**–**e**) mRNA expression analysis by qRT-PCR of mucus-related genes in the colonic mucosa. (**f**) Intestinal permeability was measured by quantitation of levels of serum FITC-Dextran (4 kDa) following oral gavage (*n* = 5 except LFD + Vehicle, *n* = 4). (**g**) Plasma lipopolysaccharide (LPS) level in Diet-Induced Obesity (DIO) mice treated with SR141716A for four weeks and controls was quantified (*n* = 5). (**h**) Myeloperoxidase (MPO) levels in colonic tissue were measured (*n* = 5). Data are shown as mean $\pm$ SD. Data with different superscript letters are significantly different (*P* < 0.05) according to post hoc ANOVA one-way statistical analysis.

SR141716A counteracted diet-induced colonic mucosal barrier dysfunction during high-fat diet feeding by modulating the mucosal thickness (Fig. 4a,b). The expression of mucus-related genes, Mucin2 (Muc2) and Kruppel-Like Factor 4 (KLF4) were increased with SR141716A treatment in Diet-Induced Obesity (DIO) mice (HFD + SR) when compared with vehicle-treated Diet-Induced Obesity (DIO) mice (HFD + Vehicle), pair-fed to SR141716A (PFSR) controls, and body weight-matched (BWM) controls (Fig. 4c,d). SR141716A did not show any effect on the Trefoil Factor 3 (Tff3) gene expression (Fig. 4e). Trefoil Factor family of secretory proteins are expressed by the gastrointestinal mucus layer. Although their functions are not clear, they are expected to be protective by stabilizing mucus layer and healing disrupted epithelium. To visualize histological changes better in the colon of different groups, the image of the whole colon is included. (Supplementary Figure 4)

The hallmark of colonic inflammation due to fat-dense diet is gut leakage and subsequently elevated levels of lipopolysaccharide (LPS) and the leukocyte enzyme myeloperoxidase. To examine the effect of the SR141716A treatment on gut integrity, we performed *in vivo* intestinal permeability assay using an FITC-labelled dextran method. Less leakage in the gut of SR141716A-treated Diet-Induced Obesity (DIO) mice (HFD + SR) was observed when compared to vehicle-treated Diet-Induced Obesity (DIO) (HFD + Vehicle) and pair-fed to SR14716A (PFSR) controls (Fig. 4f). Taken together, these data indicated that SR141716A intervention treatment in Diet-Induced Obesity (DIO) mice (HFD + SR) ameliorates the compromised mucosal layer and gut leakage in Diet-Induced Obesity (DIO) phenotype.

Additionally, our data showed that there was a significant reduction in lipopolysaccharide (LPS) levels in the serum of SR141716A-treated Diet-Induced Obesity (DIO) mice (HFD + SR) when compared to vehicle-treated Diet-Induced Obesity (DIO) (HFD + Vehicle), pair-fed Diet-Induced Obesity (DIO) control (PFSR), and body weight matched-control (BWM) mice (Fig. 4g). Local inflammation in colonic tissue was also determined by assessing the level of myeloperoxidase. SR141716A-treated Diet-Induced Obesity (DIO) mice (HFD + SR) showed significant improvement in colonic inflammation, independent of its effect on weight loss and diet intake (Fig. 4h). Based on such criteria, we propose that improvement in colon morphology following blockade of cannabinoid CB1 receptor in obese phenotype is associated with amelioration of gut inflammation.

### Effect of SR141716A on Endocannabinoid (eCB) System

**Figure 5:**
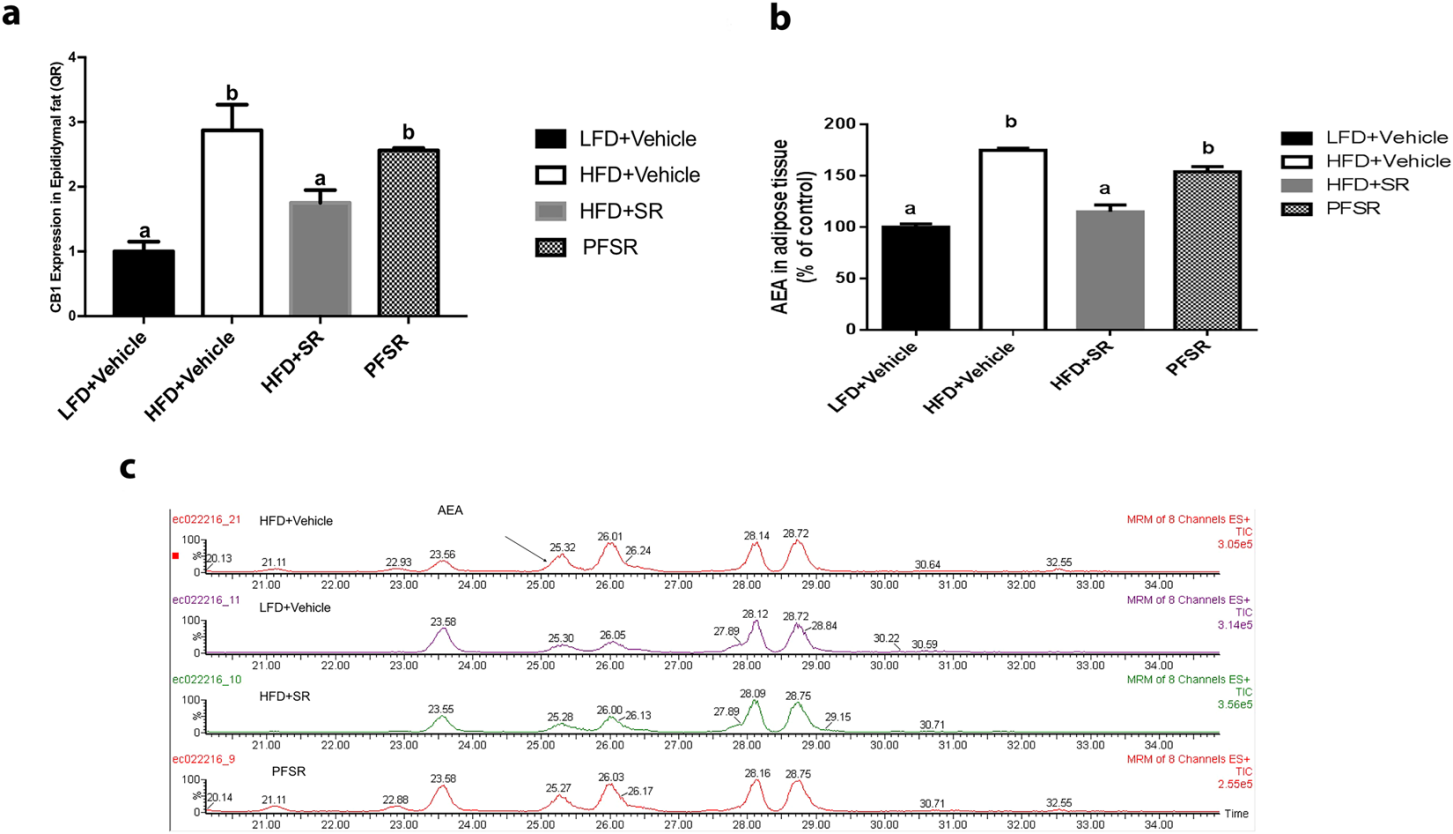
SR141716A attenuates overactivity of endocannabinoid system in diet-induced obesity. Experiments Diet-Induced Obesity (DIO) were set up as described in Fig. 1 legend. (**a**) Adipose tissue CB1 mRNA levels in SR141716A-treated Diet-Induced Obesity (DIO) (HFD + SR), vehicle-treated Diet-Induced Obesity (DIO) (HFD + Vehicle), lean mice (LFD + Vehicle) and Pair-fed to SR141716A (PFSR) control mice was assessed by RT-PCR. (**b**) White adipose tissue AEA levels from the same mice (percent of control values) were measured with Liquid Chromatograph/Mass Spectrometry/Mass Spectrometry (LC/MS/MS) (*n* = 3). N-archidonoylethanolamine (AEA) levels (percent of LFD + Vehicle) were calculated in the epididymal adipose tissue of HFD + SR, HFD + Vehicle, and PFSR (*n* = 3). Data are shown as mean $\pm$ SD. Data with different superscript letters are significantly different (*P* < 0.05) according to post hoc ANOVA one-way statistical analysis.

Obesity has been characterized by over-activation of eCB system^13^. In the current study, we found that CB1 receptor expression was down-regulated with the SR141716A treatment of Diet-Induced Obesity (DIO) mice (HFD + SR) when compared to vehicle-treated Diet-Induced Obesity (DIO) mice (HFD + Vehicle) (Fig. 5a). The level of endogenous ligands of cannabionoid receptors in adipocytes and serum was assessed by Liquid Chromatograph/Mass Spectrometry/Mass Spectrometry (LC/MS/MS). We observed a significant reduction in adipose tissue anandamide (AEA) in Diet-Induced Obesity (DIO) mice treated with SR141716A (HFD + SR) when compared to the control Diet-Induced Obesity (DIO) mice (HFD + Vehicle) (Fig. 5b,c). We were unable to detect significant levels of 2-arachidonyl glycerol (2-AG) in all samples. It is well established that Cannabinoid CB1 receptor agonists increase cannabinoid CB1 receptor activity whereas cannabinoid CB1 receptor antagonists decrease its expression^22^. While the reasons for this effect are not clear, it is believed that the use of the antagonist leads to increase in endocannabinoids that cannot act on the receptors to activate them.

### Effect of SR141716A on adipogenic related-genes

**Figure 6:**
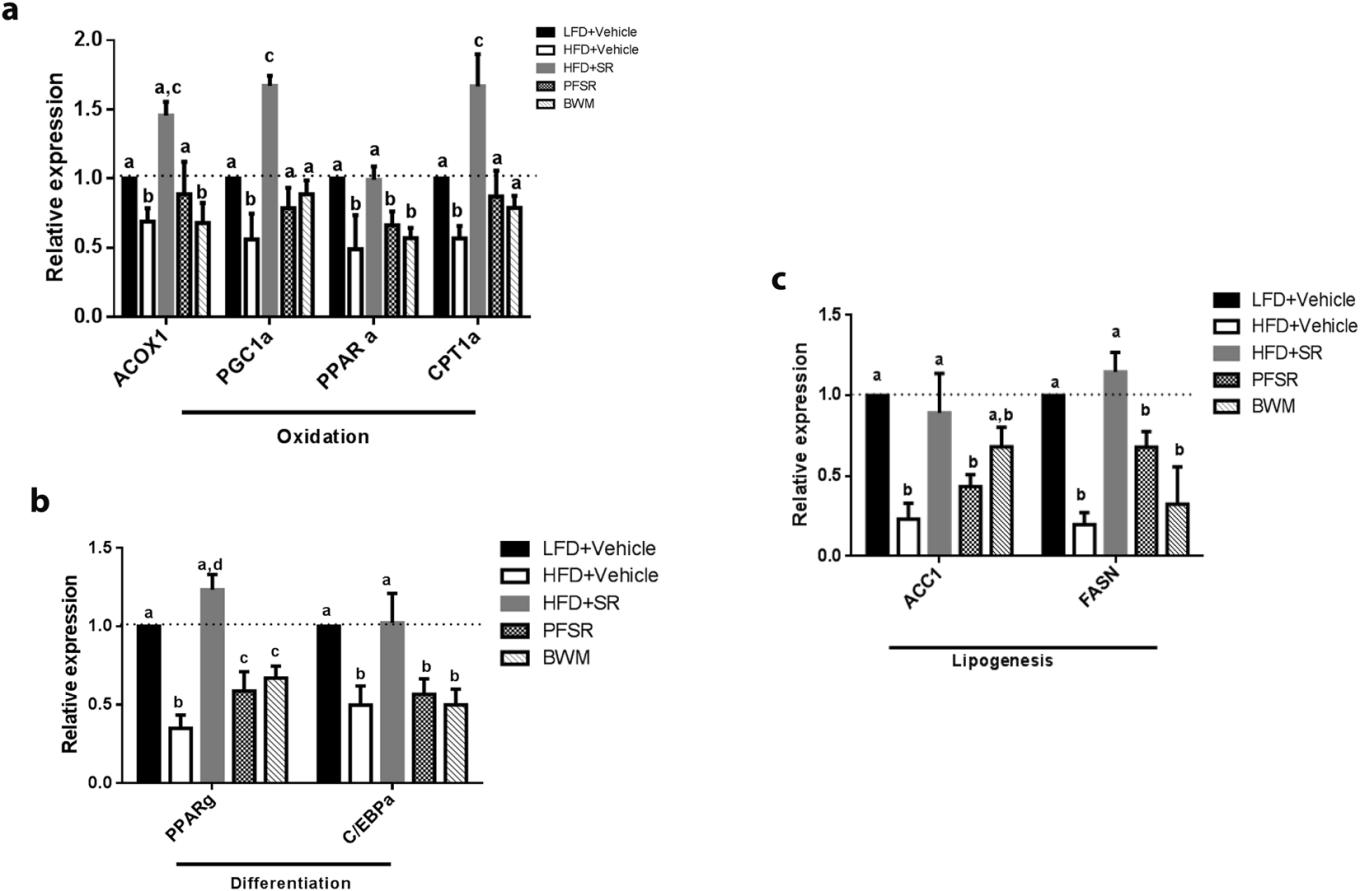
SR141716A improves adipose tissue metabolism in diet-induced obesity. Experiments Diet-Induced Obesity (DIO) were set up as described in Fig. 1 legend. mRNA expression of markers of (**a**) lipid oxidation (CPT1; ACOX1; PGC-1α; and PPARα), (**b**) adipocyte differentiation (C/EBPα, PPARγ), and (**c**) lipogenesis (ACC1; FASN) was measured in epididymal fat depots (n = 5). Data are shown as mean ± SD. Data with different superscript letters are significantly different (P < 0.05) according to post hoc ANOVA one-way statistical analysis.

Next, we investigated the effect of SR141716A on adipose tissue metabolism, which was assessed by RT-PCR for lipogenesis, oxidation and differentiation genes. We observed that SR141716A treatment of Diet-Induced Obesity (DIO) mice (HFD + SR) increased the mRNA expression of markers of lipid oxidation such as carnitine palmitoyltransferase-1 (CPT1), acyl-CoAoxidase (ACOX1), peroxisome proliferator-activated receptor gamma coactivator-1 alpha (PGC-1α), and peroxisome proliferator-activated receptor alpha (PPARα) (Fig. 6a), as well as adipocyte differentiation including CCAAT/enhancer–binding protein-α (C/EBPα)) and peroxisome proliferator-activated receptor γ (PPARγ) (Fig. 6b). Changes in lipogenic properties of adipose tissue were examined by acetyl-CoA carboxylase (ACC1) and fatty acid synthase (FASN) quantification (Fig. 6c). Together, our data suggested that the shrinkage in fat mass in SR141716-treated Diet-Induced Obesity (DIO) mice (HFD + SR) was associated with an increase in lipid oxidation differentiation and lipogenesis.

### Effect of SR141716A on dysbiosis of gut microbiota in diet-induced obesity

**Figure 7:**
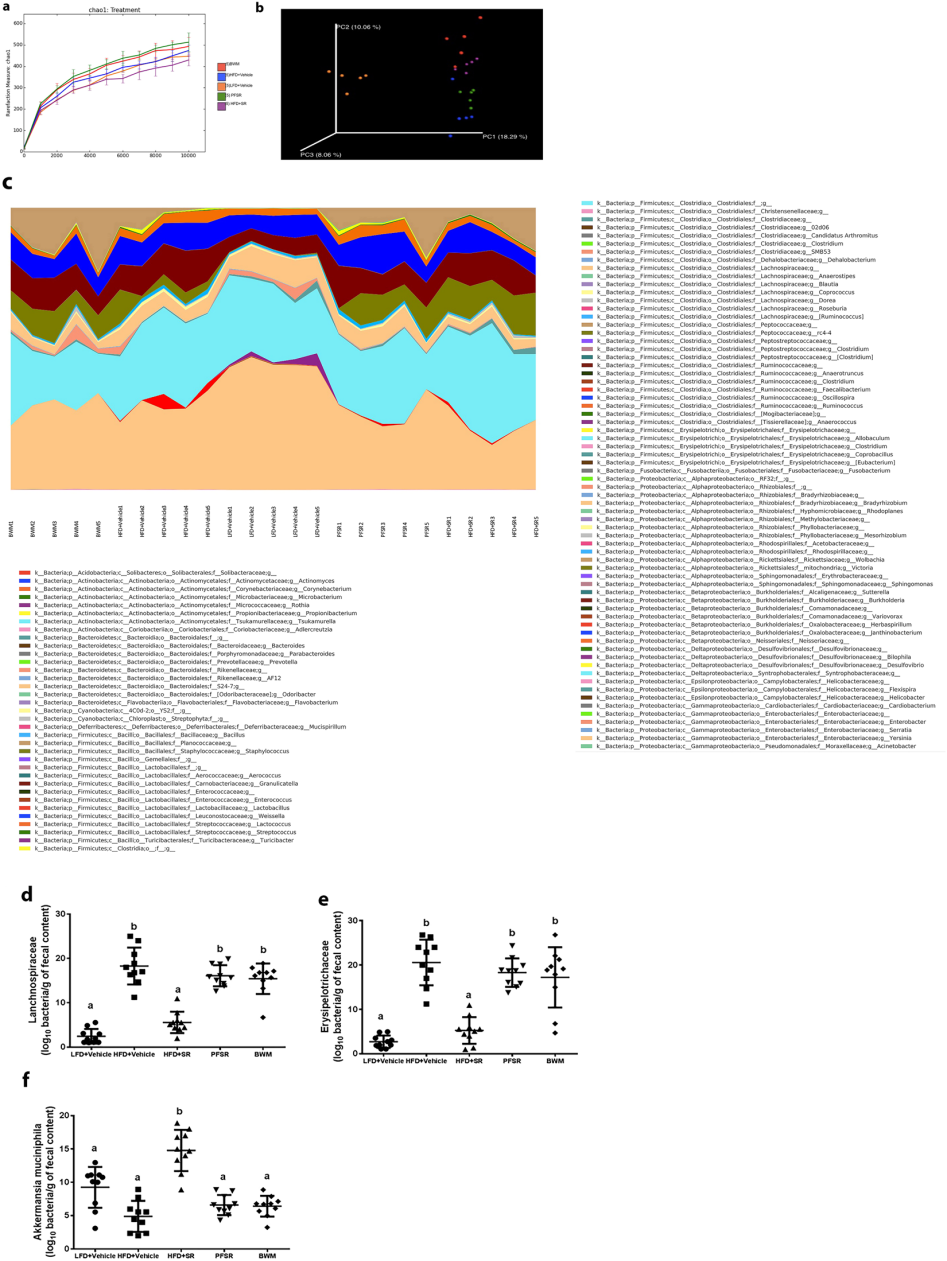
SR141716A alters gut microbiota in diet-induced obesity. Experiments Diet-Induced Obesity (DIO) were set up as described in Fig. 1 legend. Metagenomic analysis was performed on 16S rRNA V3 + V4region data, rarefied to a depth of 10,000 reads per sample. (**a**) Species richness metric based on Chao1 method was calculated. (**b**) Beta-diversity of the gut microbiome was evaluated by weighted UniFrac-based principal co-ordinate algorithim. The analysis was performed using the abundance matrix of genus-level Operational Taxonomical Units (OTUs) in different samples, and pairwise community distances were determined with 0.97 similarity using the weighted UniFrac algorithm. (**c**) Relative taxa abundance area plots for individuals from the five populations, summarized at the genus level. Individuals are represented along the horizontal axis, and relative taxa frequency is denoted by the vertical axis. (**d**) *Lanchnospiraceae* (**e**) *Erysipelotrichaceae* and (**f**) *A. muciniphila* abundance (log_10_ of bacteria per g of fecal content) was measured in mice (n = 10). Values with different superscript letters are significantly different, (*P* < 0.01) according to post hoc ANOVA one-way statistical analysis.

To test the role of gut microbiota, we performed 16S rRNA metagenomic sequencing of both variable regions (V3 + V4) of fecal samples in different groups of our study (n = 5 per group), and rarefied to a depth of 10,000 reads per sample. We arranged microorganisms in Operational Taxonomic Units (OTUs) to standardize grouping based on 97% similarities in DNA sequence (Supplementary Table 3). The data obtained demonstrated that overall, microbial communities were strongly structured by diet. Alpha diversity was calculated based on the Chao1 index to estimate the diversity of microorganisms in regards to their numbers and their similarities in abundance, (Fig. 7a). Also, beta diversity was studied by principal coordinate analysis (PCoA) wherein we observed significant separation between lean mice and Diet-Induced Obesity (DIO) mice. Interestingly, PCoA performed based on distance matrix demonstrated that microbial community structure was more sensitive to SR141716A treatment than dietary fat intake (Fig. 7b). Relative taxa abundance area plots at the genus taxonomical level for individuals from the five groups was assessed by taking the Operational Taxonomic Units (OUTs) Table at genus level as an input (Supplementary Table 4). Individual mice were represented along the horizontal axis, and related taxa frequency at the genus level was denoted by the vertical axis (Fig. 7c). Our data suggested that Operational Taxonomic Units (OTUs) were differentially enriched within the different groups. To investigate the particular effect of SR141716A on the gut-flora of Diet-Induced Obesity (DIO) mice (HFD + SR), we conducted studies with pair-fed to SR141716A (PFSR) and body-weight matched controls to Diet-Induced Obesity (DIO) mice treated with SR141716A (HFD + SR). Our data indicated significant enrichment of *Akkermansia muciniphila* OTUs in Diet-Induced Obesity-treated mice with SR141716A (HFD + SR) when compared with both pair-fed obese (PFSR) and body-weight matched (BWM) controls. Interestingly, the significant reduction in families, *Lanchnospiraceae* and *Erysipelotrichaceae* with SR141716A treatment was beyond the effect of SR141716A on weight loss and diet intake restriction. Because the disruption in gut mucosal layer was improved in Diet-Induced Obesity (DIO) mice with SR141716A treatment (HFD + SR), we investigated the effect of therapy on residential bacteria of mucosal layer, specifically *Akkermansia muciniphila* ^23,24^.

Numerous studies have shown the inverse correlation between the abundance of *A. muciniphila* and metabolic syndrome. RT-PCR from the isolated fecal DNA demonstrated significant enrichment in *A. muciniphila* colonization in SR141716A-treated DIO mice (HFD + SR) when compared to vehicle-treated Diet-Induced Obesity (DIO) (HFD + Vehicle), pair-fed to SR141716A (PFSR), and body weight matched to SR141716A (BWM) (Fig. 7f). Previous studies have demonstrated that *Lanchnospiraceae* and *Erysipelotrichaceae*, within Firmicutes phylum are implicated in gaining weight and induction of metabolic syndrome^10,25^. Our studies confirmed that *Lanchnospiraceae* and *Erysipelotrichaceae* were significantly decreased in SR141716A-treated Diet-Induced Obesity (DIO) mice (HFD + SR) when compared to vehicle-treated DIO (HFD + Vehicle) control. Also, the RT-PCR from the fecal content validated the 16s rRNA sequenced data (Fig. 7d,e).

### Effect of SR141716A on Short Chain Fatty Acid (SCFA) in Diet-Induced Obesity

**Figure 8:**
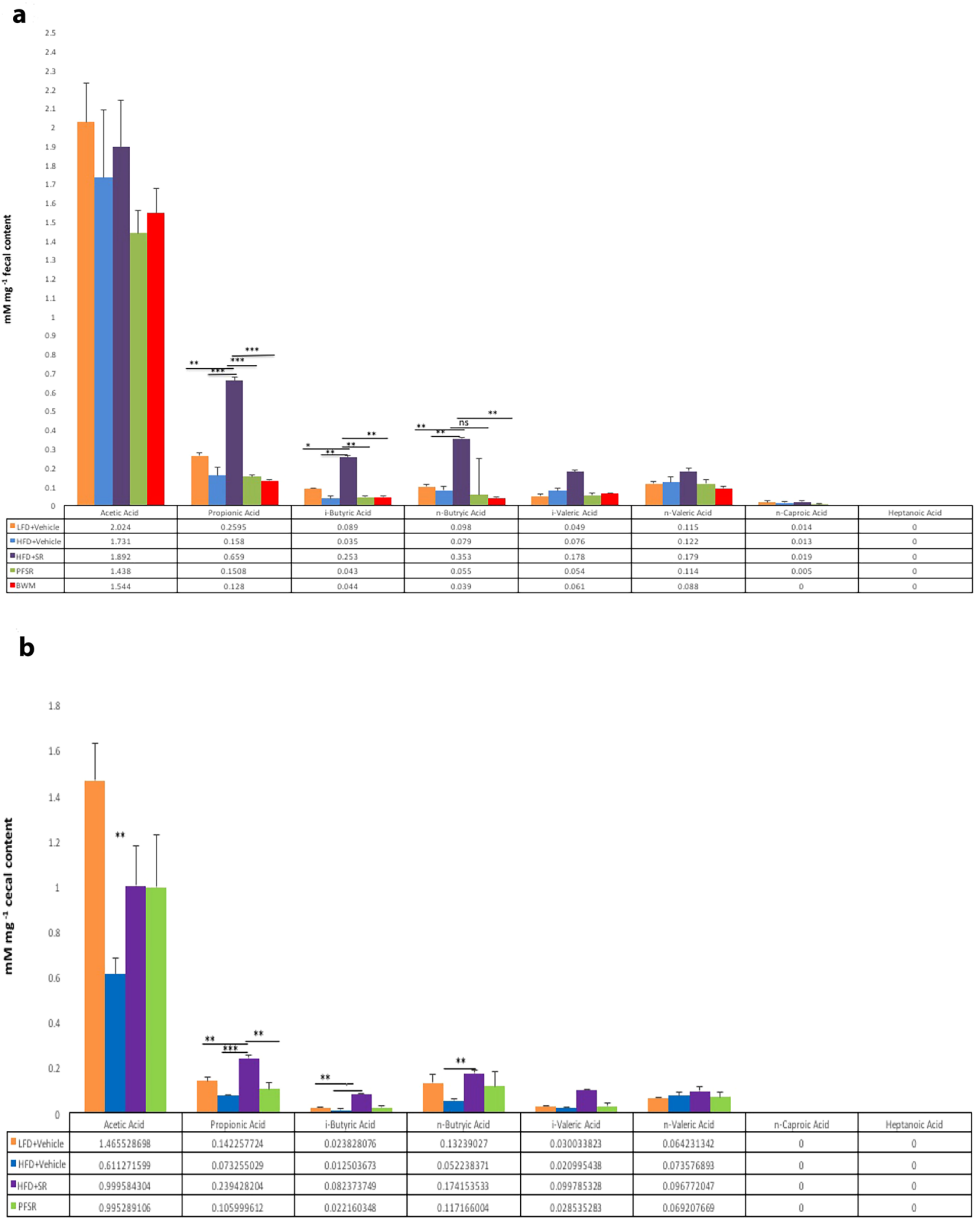
SR141716A treatment changes gut microbiome and its short chain fatty acids (SCFAs) metabolites Experiments Diet-Induced Obesity (DIO) were set up as described in Fig. 1 legend. (**a**,**b**) Gas chromatography with Flame Ionization Detector (GC-FID) quantification of short chain fatty acid (SCFA) levels in the cecal and fecal contents. Representative data are from triplicate experiments. Vertical bars represent mean ± SD. ANOVA/Tukey *p < 0.05; **p < 0.01; ***p < 0.001.

To investigate the effect of SR141716 intervention treatment in Diet-Induced Obesity (DIO) mice (HFD + SR) on short-chain fatty acids (SCFA), we quantified the level of short-chain fatty acids (SCFA) in serum, cecal and fecal content of mice. Interestingly, we found a significant increase in the concentration of propionic acid, i-butyric, as well as the n-butyric acid in the cecal and fecal content of SR141716A, treated Diet-Induced Obesity (DIO) (HFD + SR) mice when compared to vehicle-treated Diet-Induced Obesity (DIO) (HFD + Vehicle) mice (Fig. 8a,b). The same trend was observed in the concentration of acetic acid as well as valeric acid, but the changes were not significant. To evaluate the alteration in short-chain fatty acids (SCFA) systemically, we assessed the concentration of short-chain fatty acids (SCFA) in serum. Because the short-chain fatty acids (SCFAs) are mostly abundant in colon and stool, the same trend but at the lower level than short-chain fatty acids (SCFAs) in fecal and cecal content, was observed in short-chain fatty acids (SCFAs) of serum. (Supplementary Figure 5).

## Discussion

Numerous studies have demonstrated that blockade of cannabinoid CB1 receptor possesses anorectic anti-obesity properties and modulates metabolic parameters in diet-induced obesity^20,26^. However, most of the previous studies did not fully investigate the effect of cannabinoid CB1 antagonist on chronic inflammation in Diet-Induced Obesity (DIO) model. Cannabinoid CB1 receptors although expressed primarily in the brain, are also expressed in the periphery, especially in immune cells, including the gut mucosa^27^. In the current study, we examined both systemic and local inflammatory profiles in Diet-Induced Obesity (DIO) model and demonstrated that intervention treatment of Diet-Induced Obesity (DIO) mice with SR141716A, can ameliorate the obese phenotype and associated metabolic complications. Because there is a clear association between adipose tissue macrophage accumulation and metabolic dysfunction in Diet-Induced Obesity (DIO) model, we investigated the effect of CB1 antagonism on macrophages, and it was remarkable to note that SR141716A treatment could suppress pro-inflammatory macrophages (M1) in adipose tissue and their associated cytokines such as MIP-1α and MCP-1. Indeed, blockade of cannabinoid CB1 receptors in mice fed a high- fat diet reduced macrophage retention in adipose tissue, suppressed local and systemic inflammation as well as insulin resistance. In the current study, we also observed improvement in colonic inflammation (Myeloperoxidase) in Diet-Induced Obesity (DIO) model following SR141716A treatment.

It is of interest that blockade of cannabinoid CB1 receptor with SR141716A in obese mice resulted in the decrease in neutrophilic leukocytosis associated with obesity. One of the possible mechanistic effect of SR141716A on the neutrophilic leukocytosis can be attributed to the inhibition of the neutrophil elastase activity. A recent study identified that neutrophilic leukocytosis in Diet-Induced Obesity (DIO) mice exacerbates the chronic inflammation in adipose tissue^28^. The increase in neutrophil population is associated with more release of a serine proteinase, elastase, which results in activation of Toll-Like Receptor 4 (TLR4) pathway, and massive release of chemoattractants from the immune cells and adipocytes. Consistent with our findings, the neutrophilic knockout mice were protected from insulin resistance associated with obesity phenotype^28^. The direct effect of blockade of cannabinoid CB1 receptor on neutrophils and their elastase activity warrant further investigation.

To evaluate the effect of SR141716A treatment beyond its effect on calorie intake and weight loss, we conducted studies with pair-fed to SR141716A (PFSR) and body weight-matched (BWM) to SR141716A-treated obese (HFD + SR) mice as controls^29^. Pair-fed to SR141716A (PFSR) mice consumed the same amount of high-fat diet as the SR141716A-treated obese mice (HFD + SR). Diet intake was adjusted in body weight-matched (BWM) controls to perpetuate the same weight loss pattern as in SR141716A-treated obese mice (HFD + SR). Our study demonstrated that temporary reduction in calorie intake and sustained weight loss in SR141716A-treated obese mice (HFD + SR), was associated with less adiposity and smaller fat mass. The presence of smaller adipocytes in SR141716A-treated obese mice (HFD + SR) was related to the significant reduction in fat storage. Previous studies have shown that SR141716A may trigger futile calcium cycling, which results in enhanced whole body energy expenditure^20,30^. Therefore, one potential explanation for the SR141716A-induced reduction in fat mass, independent of calorie intake, is increased lipolysis and lipid oxidation to maintain ATP for the futile cycle (calcium and substrate).

In contrast to the previous studies, our data demonstrated that blockade of cannabinoid CB1 receptor with SR141716A in obese mice contribute to increased lipogenesis^29,31^. Interestingly, a previous study uncovered the regulatory role of LPS in mediating inhibitory effect on lipogenesis, on cultured adipose tissue via PPAR-γ blockade^15^. In conjunction with our data, several studies have shown that over-activity of the endocannabinoid system in Diet-Induced Obesity (DIO) model, is associated with increased LPS levels and inflammation^15^.

Furthermore, we found that SR141716A-treated obese (HFD + SR) mice demonstrated improvement in gut permeability as compared to vehicle-treated obese (HFD + Vehicle) mice. Consistent with our study, earlier reports established improvement in gut permeability in SR141716A-treated ob/ob obese mice by induction of two tight junction proteins, occludin and ZO-1^10,25^. Thus, increase in lipogenesis with SR141716A treatment may result from less gut permeability, which prevents the LPS-inhibitory effect on lipogenesis. These data also suggest that cross-talk between endocannabinoid system and lipopolysaccharide LPS may modulate adiposity.

It is exciting to note that the protective effect of SR141716A against obesity and metabolic disruption observed in our study, could be explained by the potential interactions between the gut microbial community, host metabolism, and the endocannabinoid system. However, additional studies are necessary to address if SR141716 mediates a direct effect on the microbiome or whether the changes seen in the microbiome are secondary to the effects seen on metabolism and the immune system. Enhanced endocannabinoid (eCB) system activity including a higher level of endocannabinoids in plasma and adipose tissue as well as changes in cannabinoid CB1 receptor expression has been defined in diet induced-obesity and metabolic syndrome models^13^. The CB1 receptor knockout mice are resistant to diet- induced obesity^32^. Selective reduction in CB1 receptor expression in the colon of germ-free mice, Myd88 (−/−) mice, TRIF (−/−) mice, probiotic and antibiotic treated obese mice, can be attributed to altered gut microbial composition^10,33,34^. Myd88 and TRIF are the integral adaptor molecules of toll-like receptor (TLR) signaling pathway which mediates the microbial community-host interactions^34,35^. Alteration in the gut microbial community following blockade of cannabinoid CB1 receptor in Diet-Induced Obesity (DIO) model has not been previously studied. Thus, the current study demonstrated for the first time that the protective effect of a cannabinoid CB1 receptor antagonist in Diet-Induced Obesity (DIO) is associated with restoration of gut microbial community.

Previous studies demonstrated the significant reduction in *Akkermansia muciniphila* in both genetically ob/ob and Diet-Induced Obesity (DIO) mice^36^. Protective properties of dietary polyphenols and probiotics in obese and diabetic phenotypes have been attributed to the restoration of the abundance of this strain in gut^37^. A recent study identified the protective effect of orally transferred *A. muciniphila* in dextran sulfate sodium (DSS)-induced colitis model^38^. Furthermore, adoptive transfer of live *A. muciniphila* but not the heat-killed cells were shown to ameliorate obese and diabetic phenotypes as well as reduce metabolic endotoxemia, host adiposity, and improve glucose metabolism^23^. In the present study, we demonstrated that the restored abundance of A. muciniphila in Diet-Induced Obesity (DIO) mice following blockade of the CB1 receptor was independent of calorie restriction and weight loss. To further confirm the therapeutic effect of CB1 receptor antagonist in Diet-Induced Obesity (DIO) model, we investigated the direct effect of SR141716A treatment on the colon physiology in the host. We demonstrated significant improvement in MUC2 and KLF4 genes in the colon of the SR141716A-treated obese mice when compared to the vehicle-treated obese mice. The transcription factors KLF4 and MUC2 regulate differentiation of goblet cells, which is associated with mucin formation in the colon^39^. Our data suggested that *A. muciniphila* is responding to increased host mucin production following blockade of cannabinoid CB1 receptor in Diet-Induced Obesity (DIO) mice, and mucin serves as the primary source of carbon, nitrogen, and sulfur for *A. muciniphila* growth.

Furthermore, we conducted gas chromatography to address the changes in short chain fatty acid content of the cecal material. Blockade of cannabinoid CB1 receptor caused more production of propionate and butyric acid in the cecal material. Previous studies identified the regulatory mechanism of propionate and butyrate in glucose homeostasis, lipid, and cholesterol metabolism, and improvement of gut barrier function, supporting the beneficial regulatory effect of SR141716A on the metabolic parameters in obese individuals^40^. The anti-inflammatory properties of propionate (suppression of pro-inflammatory M1 macrophages), and butyrate (inhibition of inflammation via NF-κB pathway) has been established earlier^41,42^. Consistent with our data, earlier research defined propionate as the *A. muciniphila* metabolite^35,43^. Recent studies elucidated the protective effect of propionate and butyrate against diet-induced obesity complications and metabolic syndrome. Propionate and butyrate short chain fatty acid (SCFA) have been identified to suppress appetite actively by modulating the gut hormones such as Peptide YY (PYY), and Glucagon-Like Peptide-1 (GLP-1)^24,44^. The excessive release of GLP-1 and PYY into the portal vein was identified following propionate infusion into the murine colon. Additionally, higher activity of enteroendocrine L-cells (GLP-1, and GLP-2 secretion) was identified with the growth of *A. muciniphila*, and further investigation is needed to uncover the mechanism underlying this connection^10,23^. However, whether the primary beneficial effect of SR141716A can be attributed to the gut abundance of *A. muciniphila* or higher activity of L-cells in Diet-Induced Obesity (DIO) remains an interesting question that warrants further investigation.

In summary, the current study suggests that the underlying mechanisms through which SR141716A, a CB1 antagonist, exerts its protective effect against diet-induced metabolic dysfunction may involve changes in the gut microbial community with an increase in *A. muciniphila* belonging to the family, *Verrucomicrobiaceae* and a decrease in the families, *Lanchnospiraceae* and *Erysipelotrichaceae*. While it is difficult to conclude that changes in microbiome was the underlying cause of the beneficial effect of SR141716A on obesity, the time course of changes seen in microbiome versus the clinical outcomes, suggests such a hypothesis. This observation, however, does not rule out the possibility that the effects of SR141716A on microbiome are secondary. While the direct application of *A. muciniphila* as the therapeutic intervention remains elusive because of its anaerobic growth conditions, strategies involving such bacteria may provide novel and economical approach for combating the global burden of obesity and metabolic syndrome.

## Materials and Methods

### Animals and SR141716A treatment

Diet-Induced Obesity (DIO) was studied in male C57BL/6 J mice (Jackson Laboratory, Bar Harbor, ME) by feeding the high fat diet (HFD) of 60 kcal% fat (Research Diets Inc, New Brunswick, NJ). Lean age-matched controls were fed with the low fat diet (LFD) of 10 kcal% fat, and matched with 17% sucrose in high fat diet (HFD) (Research Diets Inc, New Brunswick, NJ). High-fat diet (HFD) was started at 4 weeks of age and after 12 weeks of the high fat diet (HFD), obese mice were treated with either SR141716A or vehicle by daily oral gavage. Pair-fed and body weight-matched controls were included in the study to investigate the effect of SR141716A treatment beyond its effect on food intake and body weight loss. Pair-fed and body weight matched controls were included as previously described^29^. SR141716A was administered to the Diet-Induced Obesity (DIO) mice in 0.1% tween-80 for four weeks (10 mg/kg/daily) by daily oral gavage. Control lean (LFD + Vehicle), Diet-Induced Obesity (DIO) (HFD + Vehicle), pair-fed to SR141716A (PFSR) and body weight-matched (BWM) controls were treated with vehicle (0.1% tween-80) by daily oral gavage. The number of mice in every group and each replicate was 5–10. Body composition was assessed by using a Dual-Energy X-ray Absorptiometry (DEXA, LUNAR, Madison, WI) at the baseline of the study. Mice were normalized to the different groups based on the fat mass. Food intake was monitored daily, and changes in body weight were recorded daily after starting the intervention treatment. Mice were sacrificed under anesthesia, and different tissues were dissected. Metabolic parameters were collected at both baselines and before the sacrifice day.

### Assessment of local and systemic inflammatory profile

Cytokines levels were measured in plasma by using Bio-Plexmultiplex immunoassay system (Bio-Rad,Hercules, CA), as described by us previously^45^. RNA was isolated from epididymal fat pad using the E.Z.N.A.® Total RNA Kit (Omega Bio-Tek, Norcross, GA). The purity and concentration of the RNA were confirmed spectrophotometrically with Nanodrop (Thermo Scientific,Waltham, MA). Total RNA was converted to cDNA using the miScript cDNA synthesis kit (Qiagen, Valencia, CA) according to the manufacturer’s instructions. SsoAdvanced™ Universal SYBR® Green Supermix kit (Bio-Rad,Hercules, CA) was used to analyze gene expression, and GAPDH was used as the housekeeping gene. List of all the primers has been provided in Supplementary Table 5. Complete Blood Cell count (CBC) was performed using hematological analyzer VetScan HM5 (ABAXIS, Union City, CA). Circulating LPS level was quantified as previously described^46^. Colonic myeloperoxidase was assessed according to the manufacturer’s instruction (Abcam, Cambridge, MA)^47^. Free fatty acid was quantified in serum according to the manufacturer’s instruction (Zen-Bio Inc, Research Triangle Park, NC).

### Isolation of adipocytes and infiltrated cells in adipose tissue

Fat pads of mice were excised and placed in gentle MACS C Tubes (MACS Miltenyi Biotec, San Diego, CA) containing digestion medium (HBSS, 2 mg/ml collagenase (Sigma-Aldrich, St. Louis, MO) and 2% BSA, and homogenized by utilizing gentle MACS Dissociator (MACS Miltenyi Biotec, San Diego, CA). After incubation at 37 °C for 30 min with shaking, the cell suspension was filtered through a 100-μm filter and then spun at 1200 rpm for 10 min to separate floating adipocytes from the Stromal Vascular Fraction (SVF) pellet. The Supernatant was aspirated completely, and cells were re-suspended in FACS buffer for flow cytometry. Samples were digested until the majority of the SVF population were separated from the adipose tissue.

### Glucose and Insulin tolerance test

Glucose tolerance test was carried out as previously described^48^. After determining fasting blood glucose, each animal received a glucose gavage 1.5 g/kg body mass of glucose (25% D-glucose, Sigma, St.Louis, MO). Blood glucose levels were determined after 15, 30, 60 and 120 minutes. Insulin tolerance test was performed on unfasted animals by injecting i.p 1.5 U/kg body mass of insulin (HumilinR 100 U/ml) as previously described^49^. Blood glucose levels were assessed after 15, 30, 60 and 120 minutes. Total Cholesterol (TC), high-density lipoprotein-Cholesterol (HDL-C), low-density lipoprotein-Cholesterol (LDL-C), and triglycerides at the baseline and after intervention were quantified as previously described^50^. Homeostatic model assessment (HOMA) index was calculated as follows: insulin resistance index = fasting insulin (µU/ml)×fasting glucose (mmol/l)/22.5^51^.

### Measurement of adiposity and macrophage retention in adipose tissue

The mean adipocyte size in epididymal adipose tissue was quantified with ImageJ analysis software (National Institutes of Health, NIH) as previously described^50^. Macrophage retention in adipose tissue was quantified per 100 adipocytes by Spot Studio v1.0 Analysis Software (Advanced Cell Diagnostics, Hayward, CA).

### Mucosal layer staining and thickness

Mouse colon fixation and mounting were performed as previously described^52^. Periodic acid-Schiff was conducted according to the manufacturer’s instruction (Abcam, Cambridge, MA). The thickness of the mucosal layer was assessed by analysis software package Gene 5 (Cytation5, BioTek, Winooski, VT) and MetaMorph (MolecularDevices, Wokingham, UK). All the colon samples were collected from the sigmoid part of the colon and fixed in 4% paraformaldehyde. Hematoxylin and Eosin (H&E) staining was performed from the same samples. Histological and morphological changes in the different layers of the colon was assessed with the software package Gene 5 (Cytation 5, BioTek, Winooski). The schematic of the colon was shown for better understanding of colonic layers’ morphology (Supplementary Figure 4).

### Gut permeability *in vivo*

Mice were deprived of food and water for 4 hours. Intestinal permeability was measured after they received FITC-dextran (4 kDa; Sigma, St. Louis, MO) by oral gavage (500 mg/kg body weight, 125 mg/ml). Measurements were taken as described earlier^10^. Serial dilution of FITC–dextran in the serum was performed to generate the standard curve.

### Measurement of AEA and 2-AG in serum and tissue

Tissue lipids were extracted as described earlier^15^. Extracted lipid from serum and adipose tissue was processed as previously described^53^. The levels of endocannabinoids from tissue and serum were quantified by triple quadrupole mass spectrometer with electrospray ionization at the Mass Spectrometry Center at the Department of Chemistry and Biochemistry, University of South Carolina. Samples were introduced into Micromass Quattro-LC through a liquid chromatograph. It was used in tandem Mass Spectrometry (MS/MS) mode for qualitative and quantitative analyses.

### Microbial analysis after SR141716A intervention treatment of Diet-Induced Obesity (DIO)

16S rRNA metagenomic sequencing was performed on 25 fecal samples from high fat diet-fed mice treated with vehicle (HFD + Vehicle), SR141716A treated-high fat diet-fed (HFD + SR) mice, Pair-fed to SR141716A (PFSR) mice, body-weight matched to SR141716A (BWM) and age-matched low fat diet-fed (LFD + Vehicle) controls (n = 5 mice per group). The fecal samples were collected in cryo-tubes on day 27, and stored in −80 °C. DNA was extracted from frozen extruded feces (200 mg) using the QIAamp DNA Stool Mini Kit (Qiagen, Valencia, CA) according to the manufacturer’s instructions. Purified DNA was indexed with TrueSeqDNA PCR-free LT Library preparation kit for low-throughput studies (Illumina, San Diego, CA) according to the manufacturer’s instructions. DNA was PCR-amplified using primers for paired-end 16s community sequencing on the Illumina MiSeq platform using bacterial/archaeal primer sense 319 F/anti-sense 806 R targeting hypervariable regions V3-V4 ofthe 16S rRNA gene. Each primer was followed by a barcode identifier generated specifically for the set of primers. Phix V3 (25%) was used as a control for Illumina sequencing runs. The library was sequenced on 300 paired-end MiSeq run as previously described at Johns Hopkins Deep Sequencing and Microarray Core facility^54^.

### 16s rRNA gene sequence analysis

The sequences were preprocessed and demultiplexed with CASAVA 1.8.2 during conversion of bcl to Fastq^55^. The demultiplexed sequences were quality filtered for chimeras, using the Quantitative Insights Into Microbial Ecology (QIIME, version1.9.0) software package to avoid false diversity. Forward and reverse Illumina reads were joined using the SepPrep method (https://github.com/jstjohn/SeqPrep). We used QIIME default parameters for quality filtering as described previously^52^. Sequences were assigned to Operational Taxonomical Units (OTUs) using the closed-reference Operational Taxonomical Unit (OUT) picking protocol against the Greengenes database with a 97% threshold of pairwise identity.

Beta-diversity of the gut microbiome was evaluated by weighted UniFrac-based principal coordinates algorithm. The analysis was performed using the abundance matrix of genus-level Operational Taxonomical Units (OTUs) in different samples. Rarefaction was conducted (10,000 sequences per sample) and used to compare abundances of Operational Taxonomical Units (OTUs) across samples. Exceptions from study groups were observed. Variations in other environmental exposure and genetic factors resulted in outliers in each cluster. The Chao1 index was calculated to estimate the species richness of organisms present in the community.

Specific quantitative PCR (qPCR) targeting the employed fecal samples in 16S rRNA gene sequencing was performed using Quantifast SYBER Green PCR Kit (Bio-Rad, Hercules, CA). The abundance of *Akkermansia muciniphila* (*A. muciniphila*), *Lanchnospiraceae* and *Erysipelotrichaceae* was quantified by specific primers (Supplementary Table 5). Total microbial DNA was quantified and addressed as the endogenous control, for which we used universal bacterial primers 319 F and 806 R, the same used for 16S sequencing.

### Short-chain fatty acids quantification by gas chromatography with flame ionization detector (GC-FID)

Cecal contents (100 mg) were homogenized in 400 μl of deionized water, followed by acidification with 25% metaphosphoric acid (Sigma-Aldrich, St. Louis, MO) at a ratio of 1:5 (1 volume of acid for 5 volumes of a sample) for 30 min on ice as previously described^56^. Fatty acids were then isolated from the aqueous medium from the samples by centrifugation at 12,000 g for 15 min at 4 °C. Supernatants were then filtered over an Ultrafree MC column with a0.22 µm pore size (EMD Millipore, Billerica, MA), and elutes were stored at −80 °C until they were analyzed by gas chromatography with flame ionization detector (GC-FID).

Short-Chain Fatty Acids (SCFAs) concentrations in specimens were quantified according to a modified method as described earlier^57^. Calibration standards were prepared as aqueous stock solutions using the following fatty acids at the given concentrations: acetic, propionic, and *n*-butyric acid at 400 mM, isovaleric and valeric acid at 200 mM, isobutyric acid at 100 mM, and caproic and *n*-heptanoicacid at 50 mM. Each standard was injected to identify their retention times. Standard mixtures were prepared at several concentrations covering the range adequate for the sample concentrations. The internal standard (IS) 2-ethylbutyric acid was added at 0.30 mM to the standard mixtures as well as to each sample before injection. The standard mixtures with the IS were used to determine the response factors and linearity for each standard acid. Samples were prepared by first thawing at room temperature, placing 100 μL of the samples into a vial and adding 40 μL of acetone and 60 μL of 0.10 mMIS solution. Then the mixture was vortexed and centrifuged. The clear solution was transferred to a glass Gas Chromatography (GC) vial and used for analysis. Triplicates of samples were tested. A HP 5890 gas chromatograph configured with the flame-ionization detector (GC-FID) for analysis of volatile organic compounds was used for this assay. A stabilwax®-DA Column (fused silica) of 30 m × 0.32 mm i.d. coated with 0.50 μm film thickness was used. Helium was supplied as the carrier gas at a flow rate of 15 mL/min. The temperature was programmed to achieve the following run parameters: initial temperature 100 °C, hold for 0.5 min, ramp 20 °C/min, final temperature 250 °C maintained for 5 min. The injected sample volume for GC analysis was 1 μL splitless and the total run time was 18.0 min.

Response factors (RF) were calculated via dividing the peak areas of the responses by the respective concentrations of the standards. To quantify the peak area in terms of concentration, the relative response factor (RRF) was used. The relative response factor (RRF) was calculated using the formula RRF = RF_Standard_/RF_IS_. The concentration of the samples was calculated using the following equation, Conc. _samples_ = Peak Area_Sample_ × (Conc._IS_/Peak Area_IS_) (1/RRF).

### Statistical Analysis

Data were presented as mean ± SD. Differences between two groups were assessed using the unpaired two-tailed Student’s t-test. ANOVA followed by Newman-Keuls post hoc tests was performed to analyze differences between datasets that involved more than two groups. Generalized Estimating Equation (GEE) and Generalized Linear Mixed Model (GLMM) was carried out to fit a repeated measurement logistic regression. Trapezoidal Rule in R was used to assess Area Under the Curve (AUC) from the replicated experiments. Pearson correlation was performed in R with the cor () function in ggplot2. In the figures, data with different superscript letters were used to indicate statistically significant differences in groups (p < 0.05). Data were analyzed using GraphPad Prism version 7.00 for Windows (San Diego, CA), Excel, R a language and environment for statistic computing^58^, and IBM SPSS Statistics for Windows, Version 22.0. (Armonk, NY).

### Ethics Statement

All mice were housed at the American Association for the Accreditation of Laboratory Animal Care (AAALAC)-accredited Animal Resource Facility at the University of South Carolina, School of Medicine, Columbia, SC. All procedures were performed according to National Institutes of Health (NIH) guidelines under protocols approved by the Institutional Animal Care and Use Committee.

### Data Availability

The microbiome datasets are deposited in NCBI SRA database with the accession number: SRP124301.

## Electronic supplementary material

Supplementary Dataset
