# Supplementary material for: Blockade of CB1 cannabinoid receptor alters gut microbiota and attenuates inflammation and diet-induced obesity
Source: Sci Rep. 2017 Nov 15;7:15645. doi: 10.1038/s41598-017-15154-6 (PMC5688117; doi:10.1038/s41598-017-15154-6)
Supplement: Supplementary file 1 — Supplementary Dataset [file 41598_2017_15154_MOESM1_ESM.doc]

**Supplementary Figures and Tables**

**Blockade of CB1 cannabinoid receptor alters gut microbiota and attenuates inflammation and diet-induced obesity**

**Pegah Mehrpouya-Bahrami^1^, Kumaraswamy Naidu Chitrala^1^, Mitra S. Ganewatta^2^, Chuanbing Tang^2^, E. Angela Murphy^1^, Reilly T. Enos^1^, Kandy T. Velazquez^1^, Jamie McCellan^1^, Mitzi Nagarkatti^1^ and Prakash Nagarkatti^1,4^**

**^1^Department of Pathology, Microbiology, and Immunology, School of Medicine, ^2^Department of Chemistry and Biochemistry, University of South Carolina, Columbia, SC**

^4^Address correspondence and reprint requests to:

Prakash Nagarkatti, Ph. D.

Vice President for Research

Carolina Distinguished Professor

202 Osborne Administration Building

University of South Carolina

Columbia, SC 29208

(803) 777.5458 tel

(803) 777.5457 fax

E-mail: [prakash@mailbox.sc.edu](mailto:prakash@mailbox.sc.edu)

**
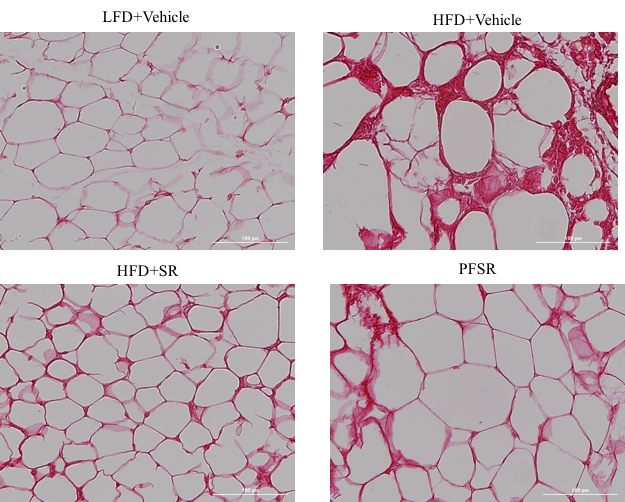
**

a

**
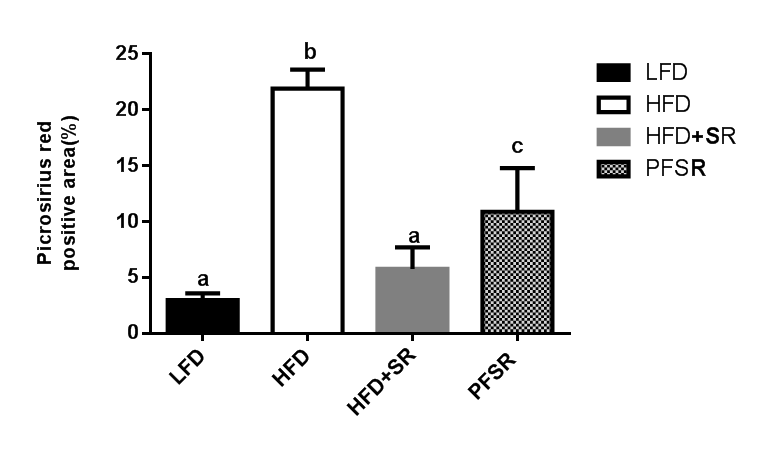
**

b

**Supplementary Figure 1.** **SR1417161A significantly reduced the interstitial fibrosis of adipose tissue in diet-induced obesity.** Experiments using Diet-Induced Obesity (DIO) mice and SR14117161 were set up as described in Fig 1 legend. (a) Representative Picrosirius Red images that were used for quantification of fibrosis area in adipose tissue of individuals (LFD+Vehicle *n=6*, HFD+Vehicle *n=10,* HFD+SR *n=9*, and PFSR *n=7*). (b) The percentage area that was Picrosirius Red-positive was quantified by converting the image to RGB (Red, Green, Blue) stack image and setting the lower and upper threshold values into the feature of interest and background in Image J software (National Institutes of Health, NIH). Data are shown as mean
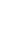
 SD. Data with different superscript letters are significantly different (*P*<0.05) according to post hoc ANOVA one-way statistical analysis.


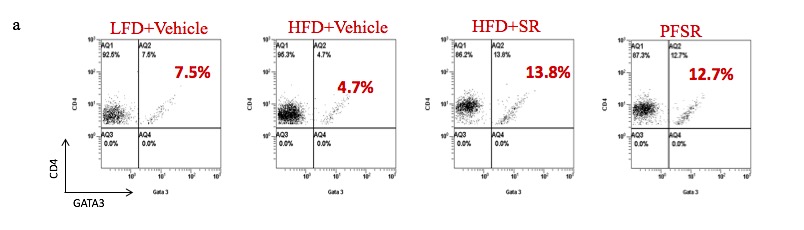


b


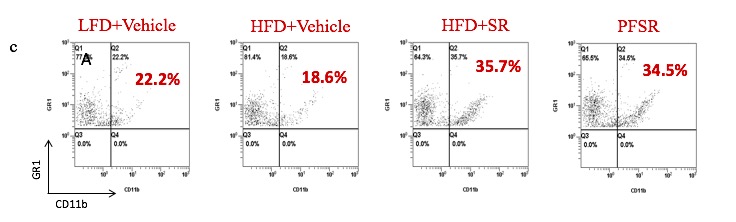


**
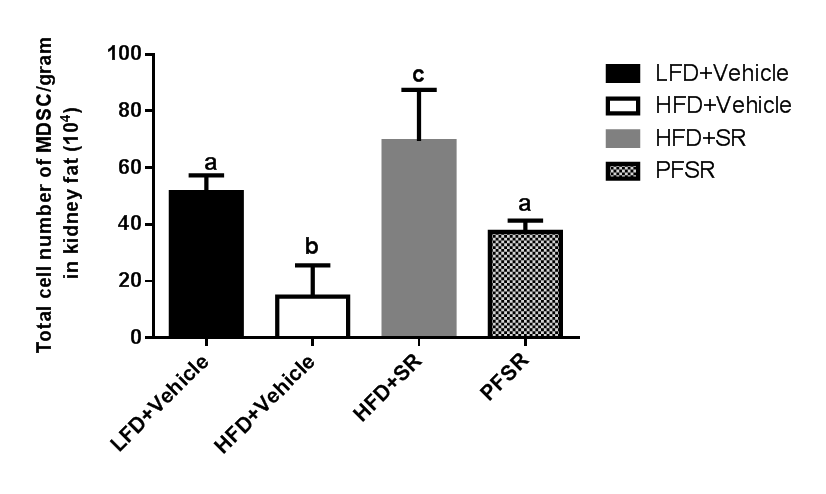
**

d

**
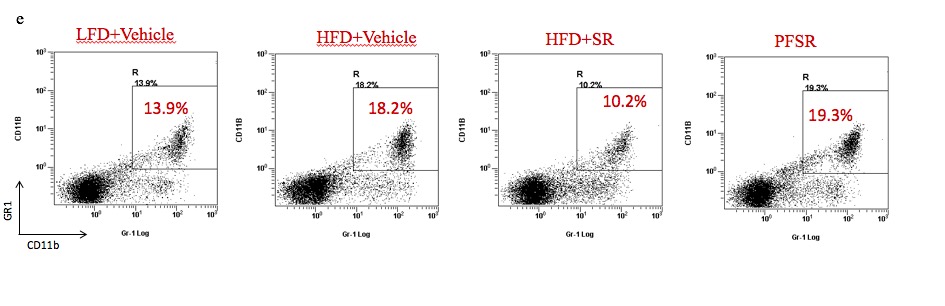
**

**Supplementary Figure** **2. SR141716A improves anti-inflammatory Th2 subset and Myeloid Derived Suppressive Cells (MDSCs) in diet-induced obesity.** (a,b) Kidney fat was isolated from 10 mice in each group. The ratio and total cell number of GATA-3+ CD4+ cells (Th2) in stromal vascular fraction of kidney fat was increased with SR141716A treatment in Diet-induced Obesity (DIO )mice (HFD+SR, 13.8%) when compared to vehicle-treated Diet-Induced Obesity (DIO) mice (HFD+Vehicle, 4.7%). (c,d) The ratio and total cell number of Gr-1+ and CD11b+ cells (MDSC) in stromal vascular fraction of kidney fat were increased with SR141716A treatment in DIO mice (HFD+SR, 35.7%) when compared to vehicle-treated DIO mice (HFD+Vehicle, 18.6%). Data are shown as mean
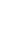
 SD. Data with different superscript letters are significantly different (*P*<0.05) according to post hoc ANOVA one-way statistical analysis. (e) The ratio of MDSC was assessed by flow cytometry in blood from different groups. The percentage of circulating GR-1+ and CD11b+ cells (MDSC) were decreased with SR141716A treatment in Diet-Induced Obesity (DIO) mice (HFD+SR,10.2%) when compared to vehicle-treated Diet-Induced Obesity (DIO) mice (HFD+Vehicle, 18.2%)

**Supplementary Table 1. SR141716A treatment improves the impaired Complete Blood Count (CBC) in diet-induced obesity.**

| **Markers#** | **LFD+Vehicle** | **HFD+Vehicle** | **HFD+SR** | **PFSR** |
| --- | --- | --- | --- | --- |
| **WBC 10^9/l** | 10.195±5.84326 | 12.6044444±6.1765729 | 10.536±5.69566151* | 8.45833333±1.86880086 |
| **LYM 10^9/l** | 5.795±4.732966 | 8.46333333±3.58347248 | 6.838±3.9607821 | 7.245±1.53385462 |
| **MON 10^9/l** | 0.48±.684377 | 0.48222222±.48625553 | 0.344±.46159626 | 0.17166667±.09495613 |
| **NEU 10^9/l** | 3.92±1.46659 | 5.6566667±3.1812576 | 3.357±2.14668763* | 1.04666667±.34621766 |
| **RBC 10^12/l** | 10.06125±.95038996 | 10.9266666666667±.63517714 | 10.41±1.00162091 | 10.415±.4429334 |
| **HGB g/dl** | 14.775±.76298287 | 15.2666666666667±.61032778 | 14.24±.962886609* | 14.6833333333333±.49564772 |
| **HCT %** | 41.3575±4.16817106 | 45.1033333333333±2.81388077 | 41.931±4.14813064* | 42.735±1.56113741 |
| **MCV fl** | 41.125±.991031209 | 41.2222222222222±.833333333 | 40.3±1.059349905 | 41±.632455532 |
| **MCH pg** | 14.775±1.41698876 | 14±.70887234 | 13.78±1.55763353 | 14.1±.4 |
| **MCHC g/dl** | 36±3.91407716 | 33.9111111111111±1.70985704 | 34.25±3.87305507 | 34.3333333333333±.46332134 |
| **RDWs fl** | 30.9375±1.23512579 | 31.5111111111111±1.08448657 | 31.55±1.74626586 | 30.8666666666667±1.45418935 |
| **RDWc %** | 18.725±.68400084 | 19.0666666666667±.4 | 19.56±1.11773581 | 18.7333333333333±.69474216 |
| **PLT 10^9/l** | 594±385.49345 | 431.222222222222±293.1850004 | 764.857142857143±542.8159383 | 773.166666666667±206.110084 |
| **MPV fl** | 5.7875±2.35519942 | 6.72222222222222±.56075346 | 6.97142857142857±.62373682 | 6.38333333333333±.38166303 |
| **PDWs fl** | 8.8±1 | 8.63333333333333±1.25830574 | 9±1.99749844 | 6.55±.27386128 |

#Whole blood was collected and subjected to the differential hematological markers: (LFD+Vehicle, *n=8*; HFD+Vehicle, *n=9;* HFD+SR, *n=10*; and PFSR, *n=6*). Data indicate mean
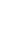
 SD. Unpaired t-test was performed between HFD+SR and HFD+Vehicle group. Statistical significance was set as value of **p* < 0.05.

**Supplementary Table 2. SR141716A treatment improves the impaired metabolic parameters in diet-induced obesity.**

| **Plasma analyses after 4-weeks treatment with SR141716A(10mg.kg^-1^.day-1) in DIO mice** | | | |  |
| --- | --- | --- | --- | --- |
| **Markers*** | **Glucose(mmol/l)** |  | **Insulin (pmol/L)** |  |
| **Weeks of Diet** | **Week12** | **Week 16** | **Week 12** | **Week 16** |
| **LFD+Vehicle** | 12.2 ± 0.7^a&^ | 12.6 ± 0.2^a&^ | 188 ± 6 ^a&^ | 190 ± 15 ^a&^ |
| **HFD+Vehicle** | 14.2 ± 0.3^a#^ | 14.8 ± 0.2 ^a#^ | 556 ± 25 ^a#^ | 634± 26 ^a#^ |
| **HFD+SR** | 14.0± 0.1^a#^ | 10.2 ± 0.4 ^b&^ | 562 ± 75 ^a#^ | 221± 46 ^b&^ |
| **PFSR** | 13.8± 0.6^a#^ | 14.2 ± 0.1 ^a#^ | 618 ± 16 ^a%^ | 490± 18b ^b%^ |
| **Markers** | **HOMA (unit)** |  | **Total Cholesterol(mmol/l)** |  |
| **Weeks of Diet** | **Week 12** | **Week 16** | **Week 12** | **Week 16** |
| **LFD+Vehicle** | 10.19 ± 0.7 ^a&^ | 10.64 ± 0.4 ^a&^ | 4.01 ± 0.01 ^a&^ | 3.99 ± 0.03 ^a&^ |
| **HFD+Vehicle** | 35.08 ± 0.5 ^a#^ | 41.7 ± 0.5 ^b#^ | 5.41 ± 0.03 ^a#^ | 6.09 ± 0.02 ^b#^ |
| **HFD+SR** | 34.96 ± 0.8 ^a#^ | 10.01 ± 0.9 ^b&^ | 5.07± 0.01 ^a#^ | 3.57 ± 0.01 ^b&^ |
| **PFSR** | 37.9 ± 1.1 ^a#^ | 30.92 ± 0.4 ^b#%^ | 5.98 ± 0.04 ^a#^ | 5.88 ± 0.05 ^a#^ |
| **Markers** | **LDL-C (mmol/l)** |  | **HDL-C (mmol/l)** |  |
| **Weeks of Diet** | **Week 12** | **Week 16** | **Week 12** | **Week 16** |
| **LFD+Vehicle** | 0.96 ± 0.02 ^a&^ | 0.98 ± 0.07 ^a#^ | 1.45 ± 0.01 ^a&^ | 1.52 ± 0.02 ^a&^ |
| **HFD+Vehicle** | 1.83 ± 0.01 ^a#^ | 1.92 ± 0.03 ^a&^ | 1.28± 0.09 ^a#^ | 1.27± 0.02 ^a#^ |
| **HFD+SR** | 1.80 ± 0.06 ^a#^ | 1.03 ± 0.02 ^b#^ | 1.34 ± 0.01 ^a&#^ | 1.83± 0.09 ^b&%^ |
| **PFSR** | 1.90 ± 0.01 ^a#^ | 1.73 ± 0.06 ^a&^ | 1.25 ± 0.07 ^a#^ | 1.27 ± 0.04 ^a#^ |
| **Markers** | **TC/HDL-C ratio (unit)** |  | **Triglycerides (mmol/l)** |  |
| **Weeks of Diet** | **Week 12** | **Week 16** | **Week 12** | **Week 16** |
| **LFD+Vehicle** | 2.6 ± 0.3 ^a&^ | 2.6 ± 0.2 ^a&^ | 0.8 ± 0.03 ^a&^ | 0.8 ± 0.05 ^a&^ |
| **HFD+Vehicle** | 4.2 ± 0.2 ^a#^ | 4.7 ± 0.9 ^a#^ | 0.9 ± 0.01 ^a#^ | 1.02 ± 0.01 ^b#^ |
| **HFD+SR** | 3.7 ± 0.8 ^a#^ | 1.9 ± 0.5 ^b&^ | 0.9 ± 0.05 ^a#^ | 0.9 ± 0.09 ^a#&^ |
| **PFSR** | 4.7 ± 0.8 ^a#^ | 4.6 ± 0.2 ^a#^ | 0.8 ± 0.04 ^a&^ | 0.9 ± 0.03 ^b&#^ |

*Plasma was assessed for fasting (5 hr) concentrations of glucose, insulin, total cholesterol (TC), high-density lipoprotein-Cholesterol (HDL-C), low-density lipoprotein-Cholesterol (LDL-C), and triglycerides prior to SR141617A treatment at week 12 of diet and at the end of the SR141716A treatment course, at week 16. Values are means ± SD (n=10, except HFD+SR n=9). Values with different superscripted letter (a,b) show significant differences over time within the group (*P*<0.05) . Values with different superscripted symbol (&#%) differ significantly among groups within the given week (*P*<0.05).

**
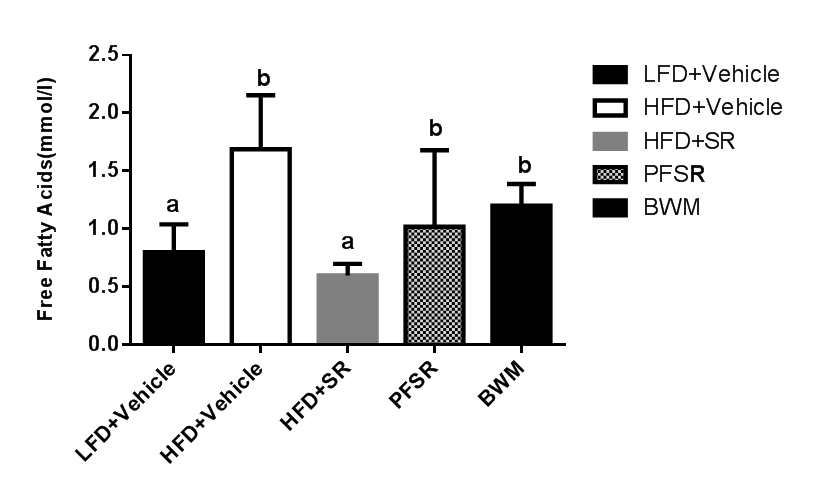

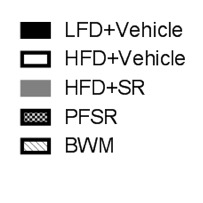
**

**Supplementary Figure 3. SR141716A treatment reverses the increase in Free Fatty Acids in diet-induced obesity.** Free Fatty Acids (FFAs) concentrations were measured in serum. Data represents mean ± SD from LFD+Vehicle (*n=5)*, HFD+Vehicle (*n=5),* HFD+SR (*n=6)*, PFSR (*n=5)*, and BWM (*n=6)*. Data with different superscript letters are significantly different (*P*<0.05) according to post hoc ANOVA one-way statistical analysis.


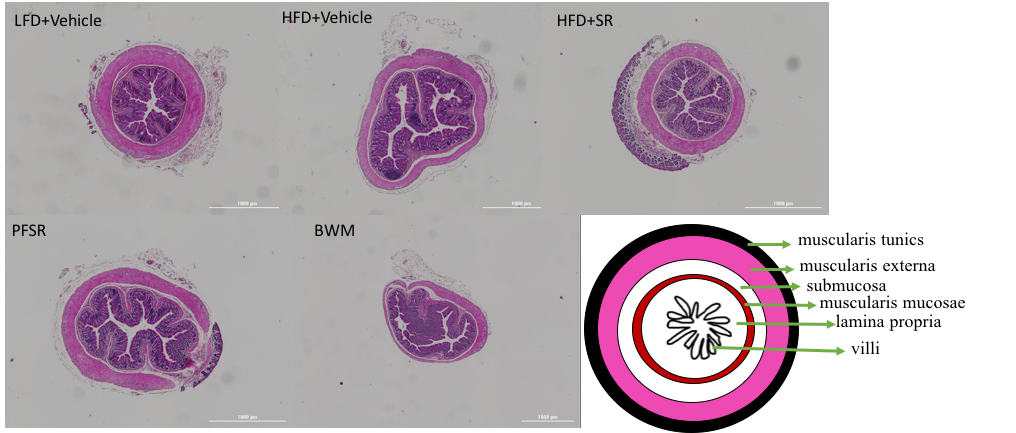


**Supplementary Figure 4. SR141716A treatment reverses the disruption of muscularis mucosae in diet-induced obesity.** Representative Hematoxylin and eosin (H&E) stain images were used for histological analysis, scale bar,1000 μm. (LFD+Vehicle *n=5*, HFD+Vehicle *n=5,* SR *n=6*, PFSR *n=5*, and BWM *n=6*).

**Table 3. Composition of Chimeras and Operational Taxonomical Units (OTUs) in 16S rRNA sequencing.**

| Observation Metadata Categories: taxonomy | | |  |
| --- | --- | --- | --- |
|  |  |  |  |
| **Sample ID:** | **OTU Counts** | **Chimeras Count** | **Chimeras%** |
| **LFD+Vehicle1** | 338281 | 39130 | 10.13% |
| **LFD+Vehicle2** | 308574 | 34101 | 9.77% |
| **LFD+Vehicle3** | 397221 | 45346 | 10.04% |
| **LFD+Vehicle4** | 330399 | 38243 | 10.20% |
| **LFD+Vehicle5** | 296668 | 31517 | 9.45% |
| **HFD+Vehicle1** | 283187 | 31372 | 9.84% |
| **HFD+Vehicle2** | 282486 | 28988 | 9.18% |
| **HFD+Vehicle3** | 313719 | 33506 | 9.52% |
| **HFD+Vehicle3** | 601577 | 64986 | 9.60% |
| **HFD+Vehicle4** | 350915 | 41863 | 10.52% |
| **HFD+SR1** | 272991 | 28203 | 9.23% |
| **HFD+SR2** | 401397 | 52916 | 11.44% |
| **HFD+SR3** | 295770 | 30971 | 9.35% |
| **HFD+SR4** | 443454 | 47524 | 9.54% |
| **HFD+SR5** | 407996 | 39303 | 8.67% |
| **PFSR21** | 235337 | 25512 | 9.61% |
| **PFSR22** | 290634 | 34094 | 10.26% |
| **PFSR23** | 220662 | 26089 | 10.33% |
| **PFSR24** | 408989 | 47556 | 10.19% |
| **PFSR25** | 280616 | 31358 | 9.91% |
| **BWM1** | 300380 | 34993 | 10.22% |
| **BWM2** | 322817 | 35825 | 9.84% |
| **BWM3** | 241940 | 27361 | 10.01% |
| **BWM4** | 314008 | 37185 | 10.44% |
| **BWM5** | 280186 | 31751 | 9.98% |

The percentage of chimeras in each Fastq files of individual samples and total counts of Operational Taxonomical Units (OTUs) was obtained from Qiime software. Number of samples: 25, Number of observations: 17245, Total count: 8220204, Table density (fraction of non-zero values): 0.219, Min counts: 220662.0, Max counts: 601577.0, Median of counts: 308574.000, Mean of counts: 328808.160 with Std. dev.:79010.459

**Table 4.** **Genus level taxonomic composition of the samples.**

[**OTUs ID**](https://docs.google.com/spreadsheets/d/e/2PACX-1vQxMxjAxCk_bcqoaQB2VMzMset-xqk0fAPyoLhMNdKEeji58rzlRY3Eu5rMTE90gJ8BOTjrhNK4TzXF/pubhtml)

Values represent the taxonomic percentage abundance of each genus in each sample.

**Supplementary Figure 5. SR141716A treatment alters Short Chain Fatty Acids (SCFAs) systemically.** Gas Chromatography with Flame Ionization Detector (GC-FID) quantification of Short Chain Fatty Acids (SCFAs) levels in serum. Representative data are from triplicate experiments. Vertical bars represent mean ± SD. ANOVA/Tukey **p* < 0.05; ***p* < 0.01; ****p* < 0.001.

**Supplementary Table 5.** **Primer sequences.** The forward and reverse sequences of each primer in the current study are provided.

Primer ID Forward Sequence Reverse Sequence
